# Quantifying Effects of Item Wording and Multiple Sources of Measurement Error Using Extended Bifactor Models

**DOI:** 10.3390/bs16091636

**Published:** 2026-09-13

**Authors:** Walter P. Vispoel, Hyeryung Lee, Tingting Chen

**Affiliations:** 1 Department of Psychological and Quantitative Foundations, University of Iowa, Iowa City, IA 52242, USA; tingting-chen@uiowa.edu; 2 School of Educational Foundations, Leadership, and Aviation, Oklahoma State University, Stillwater, OK 74076, USA; hyeryung.lee@okstate.edu

**Keywords:** reliability, item wording effects, confirmatory factor analysis, bifactor models, Self-Description Questionnaire-III, R programming

## Abstract

Researchers have long known that scores from Likert-style self-report measures of psychological traits are affected by both item wording (negative and positive) and multiple sources of measurement error (specific-factor, transient, and random-response), but the magnitude of such effects is rarely separated and individually estimated within research studies. When these effects are not distinguished, as is the case when reporting conventional reliability estimates (e.g., alpha, omega, split-half, test–retest), they are confounded with trait variance and/or each other, typically resulting in overestimation of score accuracy. We demonstrate how to quantify and separate trait, wording, and multiple measurement error effects within extended bifactor models using data from a large sample of respondents (*n* = 1796) who completed the Physical Appearance subscale from the Self-Description Questionnaire-III on two occasions. Results revealed that models including item wording effects for both single and multiple occasion designs provided noticeably superior fits to the data in comparison to models excluding such effects, with item wording accounting for 8.5% to 8.8% of total score variance across designs. We further demonstrate how to extend analyses to the individual item level to identify items that are most and least affected by wording and measurement error effects and provide code in R to enable readers to apply these techniques to their own data.

## 1. Introduction

Handling item wording effects when using Likert-style self-report measures that contain a mixture of negatively and positively worded items has long been a challenge when interpreting results from such measures. When evaluating model fit using confirmatory factor analyses, common strategies for addressing item phrasing effects include correlating uniquenesses separately for negatively and positively worded items or adding separate orthogonal (i.e., uncorrelated) factors for such items to the model. However, when applying these techniques, the magnitude of wording effects is rarely reported or separated from key sources of measurement error that affect scores at either total or individual item levels.

To set the stage for methods discussed in this article in relation to these issues and align with our illustrations focusing on measures of self-perceptions, we begin by summarizing pertinent findings from Marsh et al. (2010) involving the Rosenberg Self-Esteem Scale (RSES; Rosenberg, 1965, 1989). The goal of the Marsh et al. study was to resolve longstanding questions about the factor structure of responses to the RSES. The three most common representations over the years have been: (1) a strictly unidimensional trait model encompassing all items regardless of phrasing, (2) a model with two separate correlated factors representing negatively and positively worded items, and (3) a unidimensional trait model in which wording effects are treated as nuisance variables by correlating uniquenesses separately for negatively and positively worded items or by including separate orthogonal factors for the trait of interest, negative wording, and positive wording. As already noted, the two models within the third category are common strategies used to address wording effects when testing for model fit, but they were not used in Marsh et al. or in most other studies to estimate the magnitude of trait, wording, and multiple sources of measurement error that can affect scores.

The primary analyses reported by Marsh et al. (2010) involved data taken from the Youth in Transition study (Bachman et al., 2002). Responses to the RSES were collected in waves at four points in time: (1) early 10th grade, (2) late 11th grade, (3) late 12th grade, and (4) one year after normal high school graduation. A variety of confirmatory factor models were analyzed at each time point and across the four waves of data that included the models previously described. At each separate time point, the strictly unidimensional model provided the poorest fit (CFIs ≤ 0.767, TLIs ≤ 0.700, RMSEAs ≥ 0.089) and the unidimensional models with correlated uniquenesses (CFIs ≥ 0.987, TLIs ≥ 0.976, RMSEAs ≤ 0.020) or separate orthogonal wording factors (CFIs ≥ 0.996, TLIs ≥ 0.956, RMSEAs ≤ 0.038) provided the best fits. For the most appropriately constrained longitudinal models, the strictly unidimensional model again fit worst (CFI = 0.859, TLI = 0.844, RMSEA = 0.038), and the unidimensional model with separate orthogonal wording factors fit best (CFI = 0.982, TLI = 0.979, RMSEA = 0.014). From these results, the researchers concluded that the factor structure of RSES scores is best represented by a substantive factor representing overall self-esteem and stable response style effects related to negative and positive item wording, thereby resolving longstanding questions about the factor structure of responses to the RSES. They further speculated that similar results are likely to be found with other self-report measures of psychological traits that include negatively and positively worded items.

In this article, we use data from the Physical Appearance subscale from the adult form of the Self-Description Questionnaire (SDQ-III; Marsh, 1992) to extend the most effective and relevant models for handling wording effects described by Marsh et al. (2010) to estimate proportions of total score variance accounted for by the targeted trait of interest, negative item wording, positive item wording, and multiple sources of measurement error that can affect scores from objectively scored self-report measures. We further show how those analyses can be used to identify items most and least prone to wording and measurement error effects and provide code in R to enable readers to apply these techniques. In sections to follow, we first describe techniques for handling single-occasion data, then move to multi-occasion data, and finally describe how the analyses can be extended to the individual item score level.

## 2. Background

### 2.1. Estimating Item Wording Effects Using Single-Occasion Designs

#### 2.1.1. Designs Excluding Wording Effects

**Figure 1:**
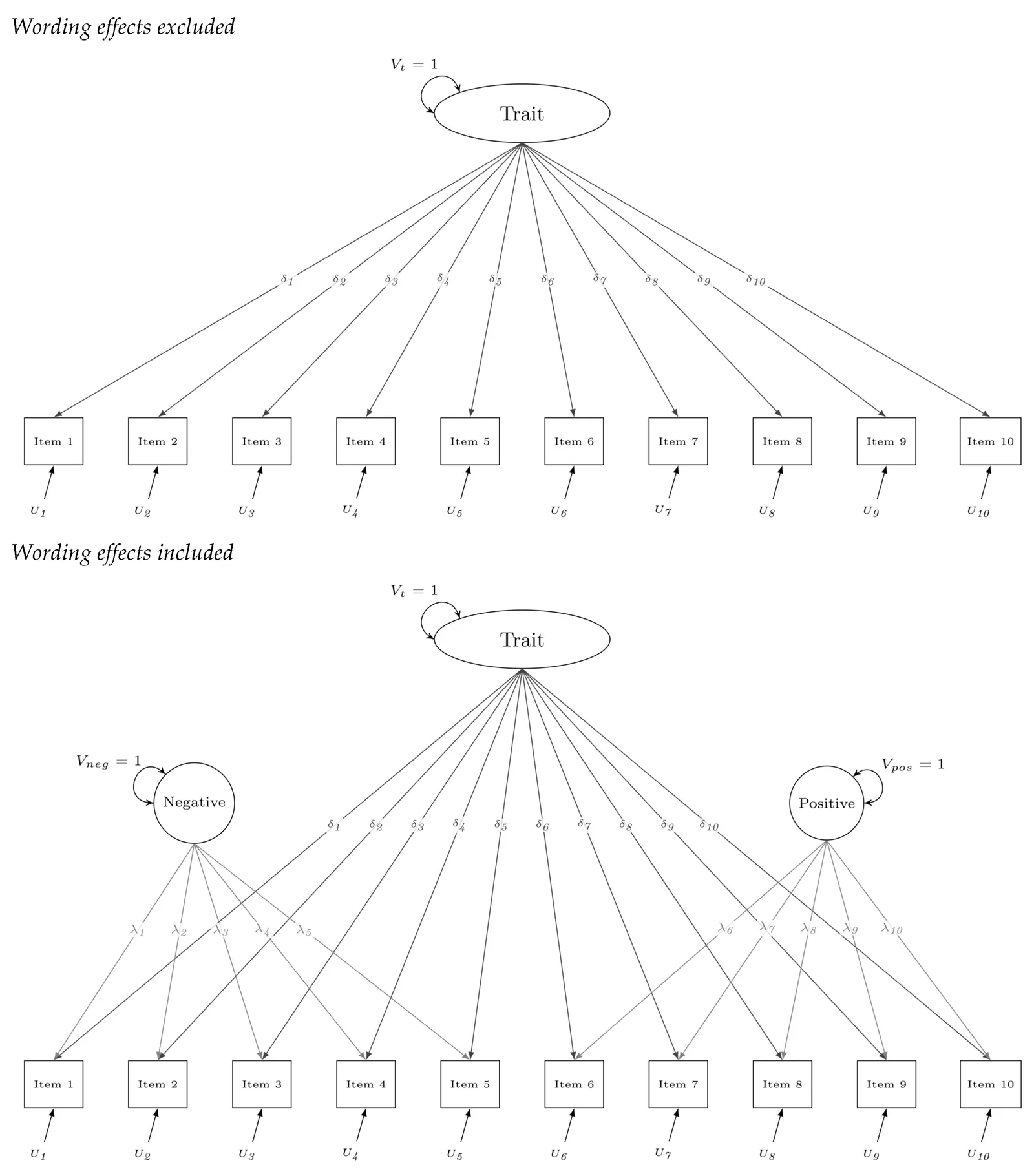
Single-occasion factor models excluding and including item wording effects. Note. $V_{t}$ = trait variance; $V_{neg}$ = negative wording factor variance; $V_{pos}$ = positive wording factor variance; $\delta_{1}-\delta_{10}$ = loadings for the trait factor; $\lambda_{1}-\lambda_{5}$ = loadings for the negative wording factor; $\lambda_{6}-\lambda_{10}$ = loadings for the positive wording factor; $U_{1}-U_{10}$ = uniquenesses.

In the top diagram in Figure 1, we depict a unidimensional factor model for a self-report measure with ten items intended to measure a single trait. All items are linked to the trait factor, whose variance is set equal to one for identification and scaling purposes. In total, 20 key parameters are estimated that together represent factor loadings and uniquenesses for all items. Once these parameters are estimated, they can be placed in formulas in Table 1 to derive variance components for the targeted trait of interest and overall measurement error. These variance components, in turn, can be placed in formulas within Table 2 to estimate proportions of total score variance accounted for by trait and overall measurement error effects.

#### 2.1.2. Designs Including Wording Effects

To extend this unidimensional trait model to account for item wording effects, separate orthogonal factors are added for parcels of negatively and positively worded items as shown in the bottom diagram in Figure 1. Only negatively phrased items are linked to the negative wording factor, and only positively worded items are linked to the positive wording factor. This results in one additional factor loading being estimated for each item, and 30 key parameters estimated within the overall model depicted with variances for the trait and each wording factor again set equal to one for purposes of identification and scaling. Once these parameters are estimated, they can be placed in formulas within Table 1 to derive variance components for the trait of interest, negative item wording, positive item wording, and overall measurement error; and these components, in turn, can be placed into formulas in Table 2 to estimate proportions of total score variance accounted for by each effect. If wording parcels vary in numbers of items, their harmonic mean is substituted within the formulas for deriving the variance components described in Table 1 and later applied in Table 2 and Table 3. Examples of computing the relevant harmonic means are included in the notes to each table.

In essence, the bottom diagram in Figure 1 represents a bifactor model (e.g., Holzinger & Harman, 1938; Holzinger & Swineford, 1937; Fang et al., 2021; Eid et al., 2018; Rodriguez et al., 2016; Vispoel et al., 2022; Vispoel et al., 2025) in which the targeted trait represents general factor effects, and types of item wording represent group factors. Such models allow for estimation of coefficients that represent proportions of variance explained by general factor effects, group factor effects, and/or both effects combined. In typical applications of bifactor models involving self-report measures, separate subscales within a trait hierarchy are analyzed with the overriding trait across subscales representing general factor effects and uncorrelated unique traits measured by each subscale representing group factor effects. In the present context, the targeted trait measured by a single subscale would represent general factor effects, and uncorrelated negatively and positively phrased item effects measured by corresponding item parcels would represent group factor effects. Total scores within such applications are equivalent to the sum or composite of item scores across the two item wording parcels. An *omega hierarchical coefficient* at the composite level would represent the proportion of total score variance accounted for by targeted trait effects alone, whereas an *omega total coefficient* would represent the proportion of total score variance accounted for by trait (general) and item wording (group) effects combined. Additional indices also can be derived to represent the proportion of total score variance accounted for solely by each type of item wording (see Table 2).

### 2.2. Estimating Item Wording Effects Within Multiple Occasion Designs

#### 2.2.1. Designs Excluding Wording Effects 

The primary limitations of single-occasion factor models are that (a) trait and state effects are confounded, and (b) key sources of measurement error are not separated and often confounded within measurement error terms. When measuring traits using objectively scored self-report measures, three sources of measurement error are most prominent: specific-factor or method effects related to items, transient error or state effects associated with occasions, and random-response and other error not captured by other effects in the model (see, e.g., Cronbach, 1947; Schmidt & Hunter, 1996; Schmidt et al., 2003; Steyer et al., 1992; Thorndike, 1951; Vispoel et al., 2018a, 2018b). In single-occasion research designs, trait and transient error effects are confounded as are specific-factor and random-response error effects, thereby typically leading to overestimates of score accuracy when reporting conventional alpha, omega, and split-half reliability estimates (Cronbach, 1947; Schmidt & Hunter, 1996; Schmidt et al., 2003; Steyer et al., 1992; Thorndike, 1951; Vispoel et al., 2018a, 2018b). To distinguish specific-factor and transient error from trait and unrelated error effects within a multiple occasion factor model design that excludes wording effects, additional item and occasion factors are added, with uniquenesses now representing error unrelated to trait, specific-factor error, or transient-error effects (i.e., random response error as described here).

**Figure 2:**
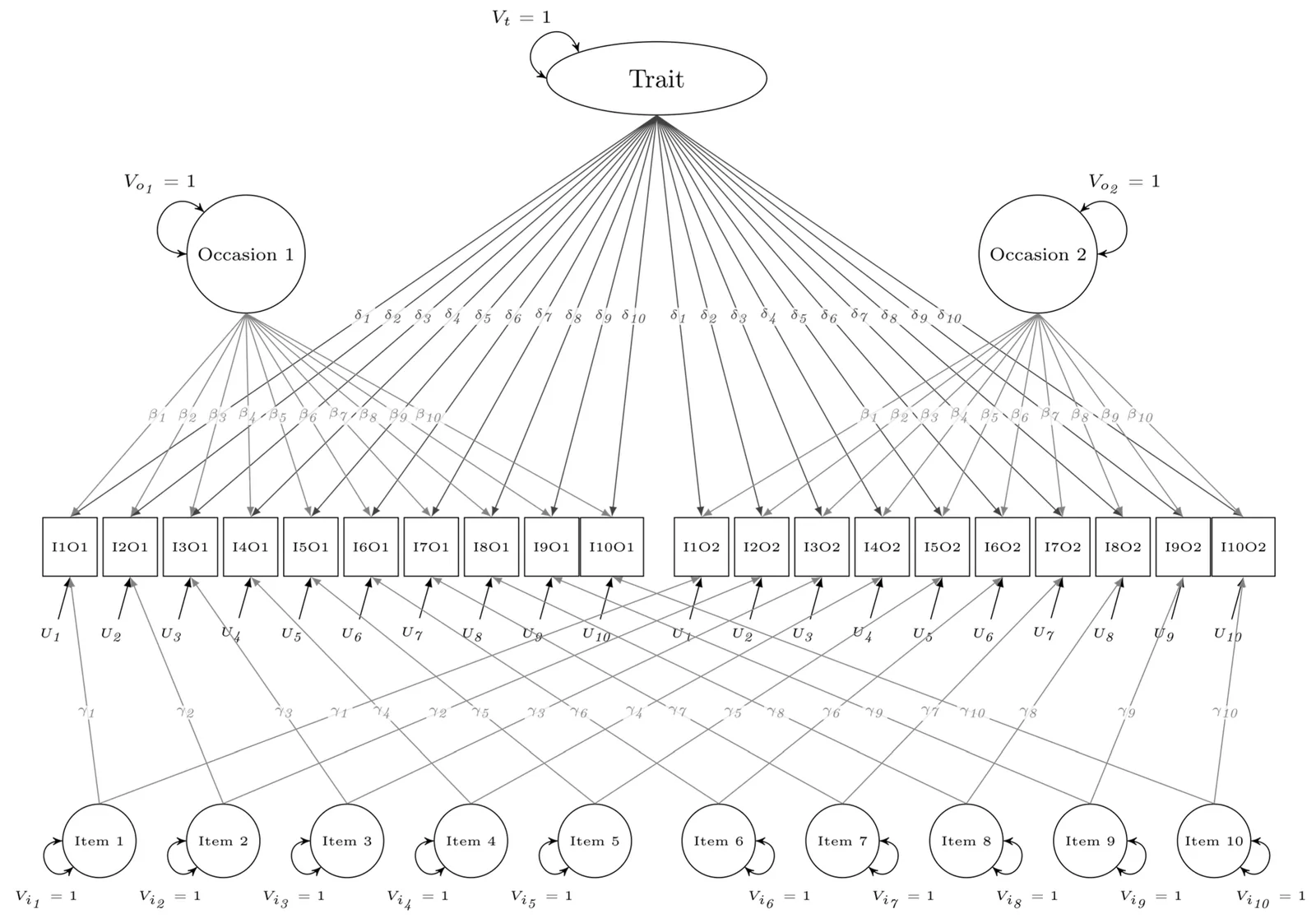
Two-occasion factor model excluding item wording effects. Note. $V_{t}$ = trait variance; $V_{o_{1}}$ = Occasion 1 factor variance; $V_{o_{2}}$ = Occasion 2 factor variance; $V_{i_{1}}-V_{i_{10}}$ = Item 1–10 factor variances; $\delta_{1}-\delta_{10}$ = loadings for the trait factor; $\beta_{1}-\beta_{10}$ = loadings for the occasion factors; $\gamma_{1}-\gamma_{10}$ = loadings for the item factors; $U_{1}-U_{10}$ = uniquenesses.

This expanded model for two-occasion data is shown in Figure 2. All items on both occasions are linked to the trait factor, and separate factors are included for each item over both occasions and for each occasion over all items. Variances for the trait, item, and occasion factors are set equal to one, and trait factor loadings, item factor loadings, occasion factor loadings, and uniquenesses are allowed to vary within an occasion but not across occasions. Altogether, 40 key parameters are estimated compared to 20 for the corresponding single-occasion model. Variance components for trait, specific-factor error, transient error, and random-response error and the associated proportions of total score variance they account for can be estimated using the formulas provided in Table 1 and Table 3.

#### 2.2.2. Designs Including Wording Effects 

**Figure 3:**
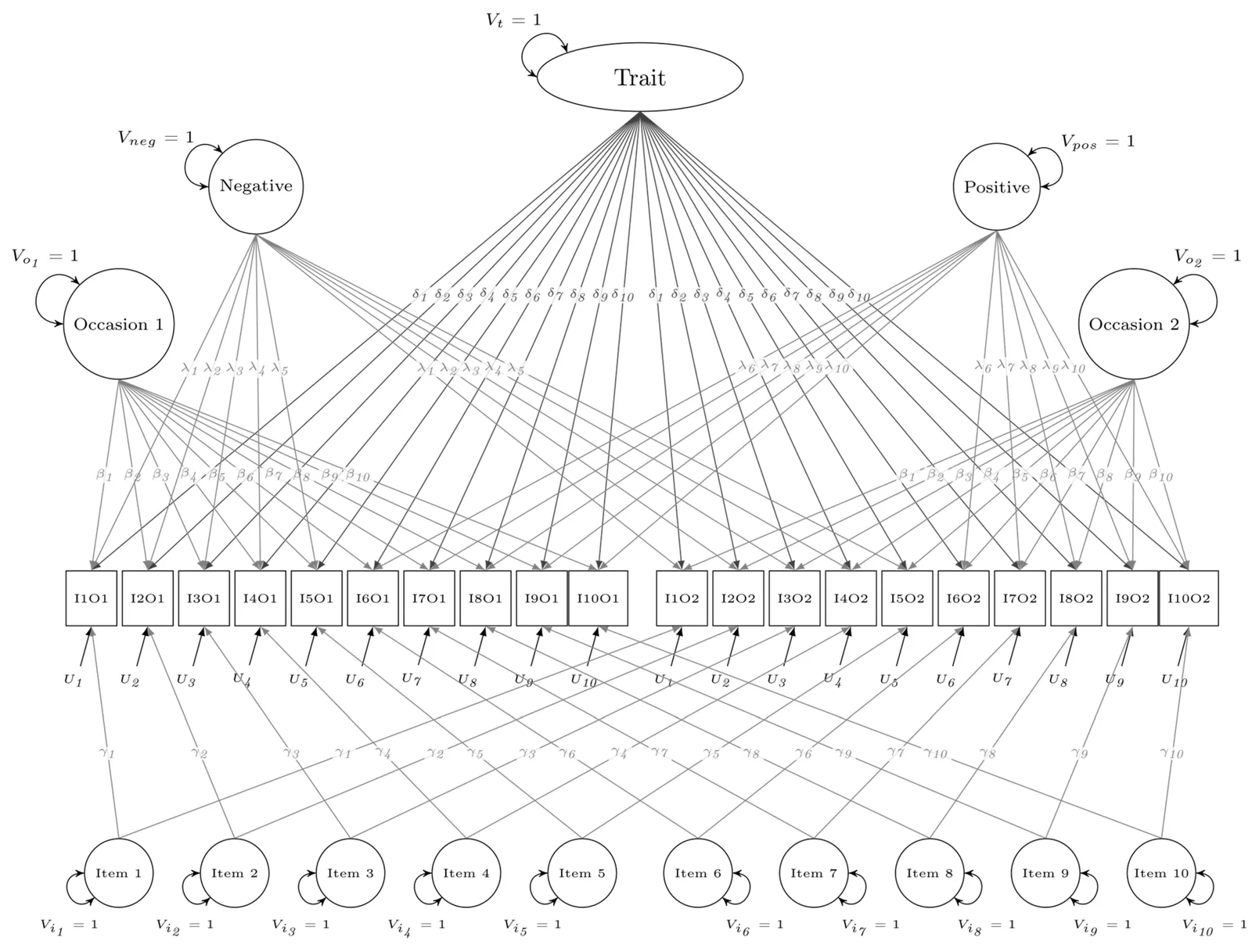
Two-occasion factor model including item wording effects. Note. $V_{t}$ = trait variance; $V_{neg}$ = negative wording factor variance; $V_{pos}$ = positive wording factor variance; $V_{o_{1}}$ = Occasion 1 factor variance; $V_{o_{2}}$ = Occasion 2 factor variance; $V_{i_{1}}-V_{i_{10}}$ = Item 1–10 factor variances; $\delta_{1}-\delta_{10}$ = loadings for the trait factor; $\lambda_{1}-\lambda_{5}$ = loadings for the negative wording factor; $\lambda_{6}-\lambda_{10}$ = loadings for the positive wording factor; $\beta_{1}-\beta_{10}$ = loadings for the occasion factors; $\gamma_{1}-\gamma_{10}$ = loadings for the item factors; $U_{1}-U_{10}$ = uniquenesses.

Extending the multi-occasion factor model design to include negative and positive item wording effects again entails adding separate orthogonal factors for each type of wording as shown in Figure 3. The negative and positive wording factors are linked only to relevantly worded items, with factor loadings allowed to vary across items but not across occasions and wording factor variances set equal to one. These modifications result in estimation of 10 additional key parameters or 50 in total compared to the previous model that excludes wording effects. Variance components for trait, item wording, and measurement error effects and the proportions of total score variance they explain again can be estimated from formulas appearing in Table 1 and Table 3.

The two-occasion design with item wording effects included again can be conceptualized as a bifactor model with the trait of interest representing general factor effects and item wording parcels representing group factor effects. However, this model is further extended to allow for separation and quantification of effects for the three primary sources of measurement error affecting scores (specific-factor, transient, and random-response). At the total score level, an omega hierarchical coefficient can be derived to represent the proportion of variance accounted for solely by trait effects, and an omega total coefficient derived to represent trait and item wording effects combined. Additional coefficients also can be derived to represent the proportion of variance accounted for by each individual wording and measurement error effect (see Table 3).

### 2.3. Partitioning Variance at the Item Score Level

Because factor loadings within the present models can vary across items, partitioning of trait, item wording, and measurement error effects can be further extended to the item level. Such analyses can be extremely valuable in the development and revision of self-report measures in choosing items that represent large proportions of trait variance and small proportions of item wording and measurement error effects. We later show how this can be done using the Physical Appearance subscale from the SDQ-III (Marsh, 1992).

## 3. Purpose

Our primary goal in this article is to demonstrate how to estimate the magnitude of trait, item wording, and measurement error effects within one- and two-occasion designs when using self-report measures with Likert-style response metrics. We also provide fit tests for all models analyzed and examples of extending partitioning of effects to the item level. When testing and evaluating the present models, we would anticipate that the addition of factors for item wording effects will improve fit for both one- and two-occasion data and that score accuracy in measuring the targeted trait in relation to its proportion of explained total score variance will decrease whenever item wording and additional measurement error effects are estimated within a given model.

## 4. Methods

### 4.1. Respondent Sample, Procedures, and Measures

We collected data from 1796 students from a large Midwestern university (73% female; 68% Caucasian; *M*_age_ = 21.52) who completed computerized versions of the Self-Description Questionnaire-III (SDQ-III; Marsh, 1992; Vispoel, 2000; Vispoel et al., 2019) on two occasions, a week apart. The one-week interval between administrations was in keeping with that used in the seminal work of Schmidt et al. (2003) into quantifying specific-factor, transient, and random-response measurement errors when using self-report measures of psychological traits. The present study was pre-approved by the university’s Institutional Review Board (IRB; ID# 200809738), and all participants provided informed consent before responding. The SDQ-III consists of 136 items that measure overall self-esteem and twelve domain-specific aspects of self-concept that primarily fall within physical, social, academic, and moral sub-domains (see, e.g., Byrne, 1996; Marsh, 1987; Shavelson et al., 1976; Vispoel, 1995). For sake of brevity, we limit the analyses reported here to the Physical Appearance subscale that includes ten items that are equally balanced for negative and positive phrasing answered along an 8-point response metric (1 = Definitely False, 8 = Definitely True). Negatively phrased items are reverse scored when computing subscale total scores. We considered the Physical Appearance subscale to be a representative measure within the SDQ-III supported by strong evidence of reliability and validity in previous research (see, e.g., Marsh, 1992; Vispoel, 2000; Vispoel et al., 2019; Byrne, 1996; Marsh, 1987; Vispoel, 1995).

### 4.2. Analyses

Preliminary analyses include estimates of subscale means, standard deviations, conventional alpha reliability coefficients (Cronbach, 1951), and conventional omega reliability coefficients (McDonald, 1970, 1978, 1999) for each occasion, as well as test–retest coefficients across occasions. The main analyses for single-occasion designs encompass model-fit tests excluding and including item wording effects and partitioning of total score variance. The corresponding procedures for the two-occasion designs include these same analyses but with separation of effects for multiple sources of measurement error. We also extend partitioning to the individual item level for designs that include trait, wording, and measurement error effects. Key analyses were conducted using the *psych* (Revelle, 2025) and *lavaan* (Rosseel, 2012; Rosseel et al., 2025) packages in R. In keeping with Marsh et al. (2010), we ran factor analyses for the one- and two-occasion designs using robust maximum likelihood estimates (MLR in *lavaan*) to anchor results to the original observed score metric that users would apply in practical applications of self-concept scores and to adjust fit indices for possible departures from normality. We considered diagonally weighted least squares (DWLS; WLSMV in R) estimation as an alternative, but that approach converts results to a hypothetical continuous, equal-interval latent-response metric rather than the original observed-score metric on which the reliability and variance-partitioning indices described here are defined. Reporting such indices as though they describe observed-score reliability has been criticized in the psychometric literature (e.g., Chalmers, 2018, on the limited usefulness of ordinal alpha). Estimation routines in generalizability theory (Cronbach et al., 1963, 1972), the framework upon which our partitioning indices extend, have historically relied on (co)variance-based estimators comparable to unweighted least squares (ULS) rather than ML-based estimators, but restricted maximum likelihood is now commonly used within that tradition as well, in part to eliminate negative variance-component estimates. These two approaches generally converge closely except in such boundary cases. We accordingly retain MLR here, consistent with Marsh et al. (2010), who used the same estimator with the RSES despite that scale having even fewer (five) response options than the 8-point format used here. (See Rhemtulla et al., 2012 for further recommendations concerning when categorical data can be treated as continuous.)

We evaluated model fit using general guidelines suggested by Hu and Bentler (1998, 1999) and Yu (2002) with adequate fits represented by comparative fit (CFI) and Tucker–Lewis (TLI) indices greater than or equal to 0.90 and root mean square errors of approximation (RMSEAs) less than or equal to 0.08, and with good to excellent fits represented by CFIs and TLIs greater than or equal to 0.95 and RMSEAs less than or equal to 0.06. Nonetheless, the suggested cut-offs serve only as rough guidelines (Marsh et al., 2004, 2005) with the fit indexes reported here being used primarily for comparative purposes in gauging the importance of integrating item wording and multiple measurement error effects into the models.

## 5. Results

We summarize the results in three ways: first, by providing descriptive statistics and conventional reliability estimates (Section 5.1); then, by comparing model fit and total-score variance partitioning for models that exclude versus include item wording effects (Section 5.2 and Section 5.3); and finally, by extending variance partitioning to the individual item level to identify items most and least affected by wording and measurement error (Section 5.2 and Section 5.3).

### 5.1. Descriptive Statistics and Conventional Reliability Estimates

Table 4 includes means, standard deviations, and conventional reliability coefficients for the Physical Appearance subscale. Using the item metric for sake of comparison, average endorsement of items is above the scale midpoint of 4.5 (*M*s = 5.21 and 5.25) with good variability (*SD*s = 1.27 and 1.30) within each occasion. Single occasion internal reliability estimates for the two occasions respectively equal 0.921 and 0.932 for alphas and equal 0.922 and 0.932 for omegas with the one-week test–retest coefficient equal to 0.925. In general, these results are highly congruent with recent studies in which computerized forms of the SDQ-III were administered on one or two occasions to similar samples of college students (see, e.g., Vispoel et al., 2019, 2023).

### 5.2. Single-Occasion Designs

Model fit indices in Table 5 reveal that the single-occasion design with wording effects provides an adequate and noticeably better fit to the data than the corresponding design excluding those effects (CFI = 0.966 vs. 0.829, TLI = 0.939 vs. 0.780, RMSEA = 0.078 vs. 0.148), thereby emphasizing the importance of systematically accounting for item wording. Total score partitioning results in Table 6 indicate that including wording effects reduces the magnitude of trait and total error effects with negative and positive wording effects, respectively, accounting for proportions of 0.044 and 0.043 of explained total score variance. To determine which items are most and least responsible for these effects, we provide item-level partitioning results in Table 7. If items with the highest proportions of trait variances coupled with lowest proportions of wording variance are considered most desirable, the results in Table 7 indicate that Items 1 and 6 are most effective in this regard, and Items 3 and 10 least effective.

### 5.3. Two-Occasion Designs

In keeping with the single-occasion designs, the fit for the two-occasion design is better when wording effects are included rather than excluded (CFI = 0.971 vs. 0.897, TLI = 0.965 vs. 0.885, RMSEA = 0.048 vs. 0.087). Accounting for measurement error effects in the two-occasion design that ignores wording effects compared to the corresponding single-occasion design without such effects does improve model fit (CFI = 0.897 vs. 0.829, TLI = 0.885 vs. 0.780, RMSEA = 0.087 vs. 0.148) but still does not reach an adequate level overall. In contrast, the two-occasion design with wording effects provides an excellent fit (CFI = 0.971, TLI = 0.965, RMSEA = 0.048). Total score partitioning for the two-occasion designs excluding and including wording effects shown in Table 8 both highlight the importance of separating and accounting for multiple sources of measurement error, with respective proportions of explained total score variance equal to 0.034 and 0.022 for specific-factor error, 0.057 and 0.044 for transient error, and 0.033 and 0.032 for random-response error. As in the previous one-occasion design, proportions of negative (0.046) and positive (0.040) wording effects on total score variance are noteworthy and serve to reduce associated proportions of trait and overall measurement error effects.

Item-level partitioning results for the two-occasion design with wording effects provided in Table 9 expand those from the associated one-occasion design to show proportions of individual sources of measurement error in addition to trait and wording effects at that level. Ideally, the best items for self-concept measures would represent large proportions of trait variance coupled with small proportions of wording and individual measurement error effects. Based on those criteria, Items 1 and 6 once again appear to be most effective for these purposes and Items 3 and 10 least effective.

## 6. Discussion

Our goal in this article was to demonstrate how to quantify trait, item wording, and measurement error effects with Likert-style self-report measures that include a mixture of negatively and positively worded items using a representative subscale from the SDQ-III (Marsh, 1992). Within both single- and multiple-occasion designs, inclusion of wording effects noticeably improved model fit while reducing proportions of trait and overall measurement error effects. In general, these findings highlight the importance of accounting for wording effects and how they are confounded with trait and/or measurement error effects when those effects are ignored. Such confounding in the present analyses of single-occasion data, in turn, led to overestimation of score accuracy primarily because transient error and item wording effects were intertwined with targeted trait effects. The results further highlighted the importance of using multiple occasion designs to (a) account for multiple sources of measurement error, (b) eliminate confounding of trait, measurement error, and wording effects, (c) provide an alternative mechanism for evaluating score reliability and construct validity, and (d) serve as a tool for choosing appropriate items in the process of developing and revising measures.

Accordingly, we encourage researchers to use the design depicted in Figure 3 whenever possible because that design allows for separation and estimation of trait, negative wording, positive wording, specific-factor error, transient error, and random-response error effects at both total and individual item score levels. Such analyses are valuable not only for providing better estimates of score accuracy but also for diagnosing the best ways to improve measures. At the total score level, specific-factor error can be reduced by adding additional items, transient error by pooling results over additional occasions, and random-response error by doing either or both. Item-level analyses allow for further precision in improving measures by identifying items most prone to wording and individual measurement error effects.

The single-occasion design represented in the bottom diagram in Figure 1 is also valuable in estimating proportions of total score variance accounted for by trait and wording effects to understand how accurately total scores measure the targeted trait and the extent to which trait effects are confounded with wording and overall measurement effects when wording effects are ignored. The main drawback to such designs is that transient error (i.e., state) effects are treated as trait variance, thereby typically inflating reliability estimates and undermining accurate interpretations of construct validity. These designs also fail to distinguish individual sources of measurement error that can provide useful insights into the best ways to improve measures.

When considering future applications, we emphasize to readers that we encountered no missing data or convergence problems within the analyses reported here. To deal with missing data, R provides a wide variety of pre-analysis procedures including *mice* (van Buuren & Groothuis-Oudshoorn, 2022), *Amelia II* (Honaker et al., 2011, 2022), and *missForest* (Stekhoven, 2022). These packages implement different multiple-imputation approaches that generate plausible values for missing observations, resulting in multiple completed datasets for subsequent analysis. If omitted responses are assumed to be missing at random, pre-analysis procedures can be avoided altogether by using full information maximum likelihood or Bayesian estimation methods.

Bifactor models in general are also sometimes prone to yielding counterintuitive results and/or non-convergence problems (Eid et al., 2017). Bayesian estimation methods again might be applied to reduce these problems (see, e.g., Vispoel et al., 2022; Muthén & Asparouhov, 2012; Liang, 2020; Liang & Yang, 2014; van de Schoot & Depaoli, 2014; Hong, 2022; Hong et al., 2024) or using scale minus one (*S* − 1) or scale indicator minus one (*SI* − 1) procedures suggested by Eid et al. (2017). The latter two procedures do reduce problems of non-convergence and counterintuitive results but alter the nature of constructs being measured and typically are not configured to subdivide measurement error into multiple sources. A more recent alternative suggested by Vispoel et al. (2026) that addresses all issues mentioned here simultaneously is to handle wording effects within a multiple occasion generalizability theory-based bifactor model framework. The primary difference between their procedures and the ones applied here is that interpretations are focused on the broader domains from which items and occasions are drawn rather than the specific ones sampled within a given study. Finally, the correlated uniqueness model strategy used by Marsh et al. (2010) described earlier also generally converges well but confounds item wording and measurement error variance (Marsh et al., 2010; Vispoel et al., 2026; Marsh & Bailey, 1991), thereby defeating the goal of the present analyses.

When reviewing the results reported here, we caution readers to interpret them in relation to the single participant sample upon which results were based that consisted of predominantly Caucasian and female college students and inclusion of scores for the single measure used for sake of illustration. These results are further bounded by our focus on a single ten-item subscale administered in an 8-point Likert-type response format with a one-week interval between occasions. Measures with different item counts, coarser or finer response scales, or different retest intervals may show varying proportions of wording and measurement-error variance, and these variations contribute to open empirical questions for future research. Although noteworthy effects were observed for both negatively and positively worded items within the present measure, such effects may not generalize to other Likert-style self-report measures that include a mixture of negatively and positively worded items. For example, in a recent follow-up study to Marsh et al. (2010) using the RSES (Rosenberg, 1965, 1989), a one-week interval between administrations, and students similar to those sampled here, Vispoel et al. (2024) found that negative wording, positive wording, specific-factor error, transient error, and random-response error, respectively, accounted for 6.3%, 0.7%, 1.9%, 7.4%, and 4.8% of total score variance in contrast to 4.6%, 4.0%, 2.2%, 4.4%, and 3.2% within the parallel design analyzed here.

When applying the techniques demonstrated here in future research, additional insights into item wording effects might be gained by examining measures with varying numbers of response options, alternative descriptors for scale points, non-proportionate representations of negative and positive phrased items, and including constructs targeted at psychological states in addition to traits. Of particular importance in such research is to apply these techniques to participant samples and measures commonly administered to younger individuals and those with weaker reading skills given that researchers have found stronger wording effects within those populations (see, e.g., Marsh, 1986; Corwyn, 2000; Schmitt & Allik, 2005; Gnambs & Schroeders, 2020; Bulut & Bulut, 2022). To facilitate such applications, we provide code in R for analyzing the present designs within our online Supplementary Materials and hope that readers will take advantage of these resources to better understand the psychometric properties of scores from self-report measures and to identify situations in which item wording effects and particular sources of measurement error are of special concern.

## Figures and Tables

**Table 1:** Formulas for estimating variance components within single and multiple occasion designs.

Design/Index	Without Wording Effects	With Wording Effects
Single Occasion		
Trait VC	${\hat{\sigma }}_{t}^{2}={\left(\frac{\sum_{i=1}^{n_{i}}{\hat{\delta }}_{i}}{n_{i}}\right)}^{2}{\;\hat{V}}_{t}\;$	${\hat{\sigma }}_{t}^{2}={\left(\frac{\sum_{i=1}^{n_{i}}{\hat{\delta }}_{i}}{n_{{\bar{H}}_{i}}}\right)}^{2}{\;\hat{V}}_{t}$
Negative Item Wording VC		${\hat{\sigma }}_{neg}^{2}={\left(\frac{\sum_{j=1}^{n_{neg}}{\hat{\lambda }}_{i}}{n_{neg}}\right)}^{2}{\;\hat{V}}_{neg}$
Positive Item Wording VC		${\hat{\sigma }}_{pos}^{2}={\left(\frac{\sum_{k=1}^{n_{pos}}{\hat{\lambda }}_{i}}{n_{pos}}\right)}^{2}{\;\hat{V}}_{pos}$
Total Measurement Error VC	${\hat{\sigma }}_{error}^{2}=\frac{\sum_{i=1}^{n_{i}}{\hat{U}}_{i}}{n_{i}}$	${\hat{\sigma }}_{error}^{2}=\frac{\sum_{i=1}^{n_{i}}{\hat{U}}_{i}}{n_{{\bar{H}}_{i}}}$
Multiple Occasions		
Trait VC	${\hat{\sigma }}_{t}^{2}={\left(\frac{\sum_{i=1}^{n_{i}}{\hat{\delta }}_{i}}{n_{i}}\right)}^{2}{\;\hat{V}}_{t}$	${\hat{\sigma }}_{t}^{2}={\left(\frac{\sum_{i=1}^{n_{i}}{\hat{\delta }}_{i}}{n_{{\bar{H}}_{i}}}\right)}^{2}{\;\hat{V}}_{t}$
Negative Item Wording VC		${\hat{\sigma }}_{neg}^{2}={\left(\frac{\sum_{j=1}^{n_{neg}}{\hat{\lambda }}_{j}}{n_{neg}}\right)}^{2}{\;\hat{V}}_{neg}$
Positive Item Wording VC		${\hat{\sigma }}_{pos}^{2}={\left(\frac{\sum_{k=1}^{n_{pos}}{\hat{\lambda }}_{k}}{n_{pos}}\right)}^{2}{\;\hat{V}}_{pos}$
Transient Error VC	${\hat{\sigma }}_{TE}^{2}={\left(\frac{\sum_{i=1}^{n_{i}}{\hat{\beta }}_{i}}{n_{i}}\right)}^{2}{\;\hat{V}}_{TE}$	${\hat{\sigma }}_{TE}^{2}={\left(\frac{\sum_{i=1}^{n_{i}}{\hat{\beta }}_{i}}{n_{{\bar{H}}_{i}}}\right)}^{2}{\;\hat{V}}_{TE}$
Specific-Factor Error VC	${\hat{\sigma }}_{SFE}^{2}=\frac{\sum_{i=1}^{n_{i}}\left({{\hat{\gamma }}_{i}}^{2}{\;\hat{V}}_{SFE}\right)}{n_{i}}$	${\hat{\sigma }}_{SFE}^{2}=\frac{\sum_{i=1}^{n_{i}}\left({{\hat{\gamma }}_{i}}^{2}{\;\hat{V}}_{SFE}\right)}{n_{{\bar{H}}_{i}}}$
Random-Response Error VC	${\hat{\sigma }}_{RRE}^{2}=\frac{\sum_{i=1}^{n_{i}}{\hat{U}}_{i}}{n_{i}}$	${\hat{\sigma }}_{RRE}^{2}=\frac{\sum_{i=1}^{n_{i}}{\hat{U}}_{i}}{n_{{\bar{H}}_{i}}}$

Note. *t* = trait; *i* = items; *j =* negatively phrased items; *k =* positively phrased items; *o* = occasions; VC = variance component; SFE = specific-factor error; TE = transient error; RRE = random-response error; $n_{i}$ = number of total items; $n_{neg}$ = number of negatively phrased items; $n_{pos}$ = number of positively phrased items; $n_{{\bar{H}}_{i}}$ = harmonic mean of $n_{neg}$ and $n_{pos}$; $\hat{V}$ = factor variance; $\hat{\delta }$ = estimated factor loading for the trait factor; ${\hat{\lambda }}_{neg}$ = estimated factor loading for the negative item wording factor; ${\hat{\lambda }}_{pos}=\mathrm{estimated}\mathrm{factor}\mathrm{loading}\mathrm{for}\mathrm{the}\mathrm{positive}\mathrm{item}\mathrm{wording}\mathrm{factor};\;\hat{\gamma }\;$= estimated factor loading for an item factor; $\hat{\beta }\;$= estimated factor loading for an occasion factor. The harmonic mean for numbers of items across item wording parcels ($n_{{\bar{H}}_{i}}^{\prime }$) equals the number of parcels divided by the sum of the reciprocals of the number of items within each parcel. For example, if there are two parcels with four and six items, the harmonic mean would equal 2/(1/4 + 1/6) or 4.8. For the present factor model designs, ${\hat{V}}_{p},$${\;\hat{V}}_{neg},{\;\hat{V}}_{pos}$, ${\;\hat{V}}_{SFE}$ and ${\;\hat{V}}_{TE}$ are set equal to 1 for purposes of identification and scaling. The formulas presented for conditions with wording effects assume an equal number of positively and negatively phrased items. If the number of items in each category differs, each component (e.g., loading, variance, or uniqueness) should be proportionally adjusted based on the relative number of positively and negatively phrased items in the total set.

**Table 2:** Formulas for partitioning proportions of total score variance within single-occasion designs.

Index/Design	Formula	
	Without Item Wording	With Item Wording
$\hat{\omega }$ total coefficient	$\frac{{\hat{\sigma }}_{t}^{2}}{{\hat{\sigma }}_{t}^{2}+\frac{{\hat{\sigma }}_{total\;error}^{2}}{n_{i}^{\prime }}}$	$\frac{{\hat{\sigma }}_{t}^{2}+{\hat{\sigma }}_{neg}^{2}+{\hat{\sigma }}_{pos}^{2}}{{\hat{\sigma }}_{t}^{2}+{\hat{\sigma }}_{neg}^{2}+{\hat{\sigma }}_{pos}^{2}+\frac{{\hat{\sigma }}_{total\;error}^{2}}{n_{{\bar{H}}_{i}}^{\prime }}}$
$\hat{\omega }$ hierarchical coefficient		$\frac{{\hat{\sigma }}_{t}^{2}}{{\hat{\sigma }}_{t}^{2}+{\hat{\sigma }}_{neg}^{2}+{\hat{\sigma }}_{pos}^{2}+\frac{{\hat{\sigma }}_{total\;error}^{2}}{n_{{\bar{H}}_{i}}^{\prime }}}$
Negative wording proportion		$\frac{{\hat{\sigma }}_{neg}^{2}}{{\hat{\sigma }}_{t}^{2}+{\hat{\sigma }}_{neg}^{2}+{\hat{\sigma }}_{pos}^{2}+\frac{{\hat{\sigma }}_{total\;error}^{2}}{n_{{\bar{H}}_{i}}^{\prime }}}$
Positive wording proportion		$\frac{{\hat{\sigma }}_{pos}^{2}}{{\hat{\sigma }}_{t}^{2}+{\hat{\sigma }}_{neg}^{2}+{\hat{\sigma }}_{pos}^{2}+\frac{{\hat{\sigma }}_{total\;error}^{2}}{n_{{\bar{H}}_{i}}^{\prime }}}$
Total wording proportion		$\frac{{\hat{\sigma }}_{neg}^{2}+{\hat{\sigma }}_{pos}^{2}}{{\hat{\sigma }}_{t}^{2}+{\hat{\sigma }}_{neg}^{2}+{\hat{\sigma }}_{pos}^{2}+\frac{{\hat{\sigma }}_{total\;error}^{2}}{n_{{\bar{H}}_{i}}^{\prime }}}$
Total error proportion	$\frac{\frac{{\hat{\sigma }}_{total\;error}^{2}}{n_{i}^{\prime }}}{{\hat{\sigma }}_{t}^{2}+\frac{{\hat{\sigma }}_{total\;error}^{2}}{n_{i}^{\prime }}}$	$\frac{\frac{{\hat{\sigma }}_{total\;error}^{2}}{n_{{\bar{H}}_{i}}^{\prime }}}{{\hat{\sigma }}_{t}^{2}+{\hat{\sigma }}_{neg}^{2}+{\hat{\sigma }}_{pos}^{2}+\frac{{\hat{\sigma }}_{total\;error}^{2}}{n_{{\bar{H}}_{i}}^{\prime }}}$

Note. *t* = trait; *i* = items; *o* = occasion; $n_{neg}^{\prime }$ = the number of negatively phrased items; $n_{pos}^{\prime }$ = the number of positively phrased items; $n_{i}^{\prime }$ = the number of total items; $n_{\bar{H}}^{\prime }$ = harmonic mean of $n_{neg}^{\prime }$ and $n_{pos}^{\prime }$. In the single-occasion designs: ${\hat{\sigma }}_{t}^{2}$ = trait variance; ${\hat{\sigma }}_{neg}^{2}=negative\;wording\;variance;{\hat{\sigma }}_{pos}^{2}\;=positive\;wording\;variance;\frac{{\hat{\sigma }}_{total\;error}^{2}}{n_{i}^{\prime }}$ or $\frac{{\hat{\sigma }}_{total\;error}^{2}}{n_{{\bar{H}}_{i}}^{\prime }}$ = total error variance. The harmonic mean for numbers of items across item wording parcels ($n_{{\bar{H}}_{i}}^{\prime }$) equals the number of parcels divided by the sum of the reciprocals of the number of items within each parcel. For example, if there are two parcels with four and six items, the harmonic mean would equal 2/(1/4 + 1/6) or 4.8. Primes are placed above the symbol *n* in the formulas to indicate that they can be altered for estimating the magnitude of indices when changing numbers of items. The variance components shown in the table are based on those defined in Table 1.

**Table 3:** Formulas for partitioning proportions of total score variance within multiple-occasion designs.

Index/Design	Formula	
	Without Item Wording	With Item Wording
$\hat{\omega }$ total coefficient	$\frac{{\hat{\sigma }}_{t}^{2}}{{\hat{\sigma }}_{t}^{2}+\frac{{\hat{\sigma }}_{SFE}^{2}}{n_{i}^{\prime }}+\frac{{\hat{\sigma }}_{TE}^{2}}{n_{o}^{\prime }}+\frac{{\hat{\sigma }}_{RRE}^{2}}{{n_{i}^{\prime }n}_{o}^{\prime }}}$	$\frac{{\hat{\sigma }}_{t}^{2}+{\hat{\sigma }}_{neg}^{2}+{\hat{\sigma }}_{pos}^{2}}{{\hat{\sigma }}_{t}^{2}+{\hat{\sigma }}_{neg}^{2}+{\hat{\sigma }}_{pos}^{2}+\frac{{\hat{\sigma }}_{SFE}^{2}}{n_{{\bar{H}}_{i}}^{\prime }}+\frac{{\hat{\sigma }}_{TE}^{2}}{n_{o}^{\prime }}+\frac{{\hat{\sigma }}_{RRE}^{2}}{n_{{\bar{H}}_{i}}^{\prime }n_{o}^{\prime }}}$
$\hat{\omega }$ hierarchical coefficient		$\frac{{\hat{\sigma }}_{t}^{2}}{{\hat{\sigma }}_{t}^{2}+{\hat{\sigma }}_{neg}^{2}+{\hat{\sigma }}_{pos}^{2}+\frac{{\hat{\sigma }}_{SFE}^{2}}{n_{{\bar{H}}_{i}}^{\prime }}+\frac{{\hat{\sigma }}_{TE}^{2}}{n_{o}^{\prime }}+\frac{{\hat{\sigma }}_{RRE}^{2}}{n_{{\bar{H}}_{i}}^{\prime }n_{o}^{\prime }}}$
Negative wording proportion		$\frac{{\hat{\sigma }}_{neg}^{2}}{{\hat{\sigma }}_{t}^{2}+{\hat{\sigma }}_{neg}^{2}+{\hat{\sigma }}_{pos}^{2}+\frac{{\hat{\sigma }}_{SFE}^{2}}{n_{{\bar{H}}_{i}}^{\prime }}+\frac{{\hat{\sigma }}_{TE}^{2}}{n_{o}^{\prime }}+\frac{{\hat{\sigma }}_{RRE}^{2}}{n_{{\bar{H}}_{i}}^{\prime }n_{o}^{\prime }}}$
Positive wording proportion		$\frac{{\hat{\sigma }}_{pos}^{2}}{{\hat{\sigma }}_{t}^{2}+{\hat{\sigma }}_{neg}^{2}+{\hat{\sigma }}_{pos}^{2}+\frac{{\hat{\sigma }}_{SFE}^{2}}{n_{{\bar{H}}_{i}}^{\prime }}+\frac{{\hat{\sigma }}_{TE}^{2}}{n_{o}^{\prime }}+\frac{{\hat{\sigma }}_{RRE}^{2}}{n_{{\bar{H}}_{i}}^{\prime }n_{o}^{\prime }}}$
Total wording proportion		$\frac{{\hat{\sigma }}_{neg}^{2}+{\hat{\sigma }}_{pos}^{2}}{{\hat{\sigma }}_{t}^{2}+{\hat{\sigma }}_{neg}^{2}+{\hat{\sigma }}_{pos}^{2}+\frac{{\hat{\sigma }}_{SFE}^{2}}{n_{{\bar{H}}_{i}}^{\prime }}+\frac{{\hat{\sigma }}_{TE}^{2}}{n_{o}^{\prime }}+\frac{{\hat{\sigma }}_{RRE}^{2}}{n_{{\bar{H}}_{i}}^{\prime }n_{o}^{\prime }}}$
Specific-factor error proportion	$\frac{\frac{{\hat{\sigma }}_{SFE}^{2}}{n_{i}^{\prime }}}{{\hat{\sigma }}_{t}^{2}+\frac{{\hat{\sigma }}_{SFE}^{2}}{n_{i}^{\prime }}+\frac{{\hat{\sigma }}_{TE}^{2}}{n_{o}^{\prime }}+\frac{{\hat{\sigma }}_{RRE}^{2}}{n_{i}^{\prime }n_{o}^{\prime }}}$	$\frac{\frac{{\hat{\sigma }}_{SFE}^{2}}{n_{{\bar{H}}_{i}}^{\prime }}}{{\hat{\sigma }}_{t}^{2}+{\hat{\sigma }}_{neg}^{2}+{\hat{\sigma }}_{pos}^{2}+\frac{{\hat{\sigma }}_{SFE}^{2}}{n_{{\bar{H}}_{i}}^{\prime }}+\frac{{\hat{\sigma }}_{TE}^{2}}{n_{o}^{\prime }}+\frac{{\hat{\sigma }}_{RRE}^{2}}{n_{{\bar{H}}_{i}}^{\prime }n_{o}^{\prime }}}$
Transient error proportion	$\frac{\frac{{\hat{\sigma }}_{TE}^{2}}{n_{o}^{\prime }}}{{\hat{\sigma }}_{t}^{2}+\frac{{\hat{\sigma }}_{SFE}^{2}}{n_{i}^{\prime }}+\frac{{\hat{\sigma }}_{TE}^{2}}{n_{o}^{\prime }}+\frac{{\hat{\sigma }}_{RRE}^{2}}{n_{i}^{\prime }n_{o}^{\prime }}}$	$\frac{\frac{{\hat{\sigma }}_{TE}^{2}}{n_{o}^{\prime }}}{{\hat{\sigma }}_{t}^{2}+{\hat{\sigma }}_{neg}^{2}+{\hat{\sigma }}_{pos}^{2}+\frac{{\hat{\sigma }}_{SFE}^{2}}{n_{{\bar{H}}_{i}}^{\prime }}+\frac{{\hat{\sigma }}_{TE}^{2}}{n_{o}^{\prime }}+\frac{{\hat{\sigma }}_{RRE}^{2}}{n_{{\bar{H}}_{i}}^{\prime }n_{o}^{\prime }}}$
Random-response error Proportion	$\frac{\frac{{\hat{\sigma }}_{RRE}^{2}}{{n_{i}^{\prime }n}_{o}^{\prime }}}{{\hat{\sigma }}_{t}^{2}+\frac{{\hat{\sigma }}_{SFE}^{2}}{n_{i}^{\prime }}+\frac{{\hat{\sigma }}_{TE}^{2}}{n_{o}^{\prime }}+\frac{{\hat{\sigma }}_{RRE}^{2}}{{n_{i}^{\prime }n}_{o}^{\prime }}}$	$\frac{\frac{{\hat{\sigma }}_{RRE}^{2}}{n_{{\bar{H}}_{i}}^{\prime }n_{o}^{\prime }}}{{\hat{\sigma }}_{t}^{2}+{\hat{\sigma }}_{neg}^{2}+{\hat{\sigma }}_{pos}^{2}+\frac{{\hat{\sigma }}_{SFE}^{2}}{n_{{\bar{H}}_{i}}^{\prime }}+\frac{{\hat{\sigma }}_{TE}^{2}}{n_{o}^{\prime }}+\frac{{\hat{\sigma }}_{RRE}^{2}}{n_{{\bar{H}}_{i}}^{\prime }n_{o}^{\prime }}}$
Total error proportion	$\frac{\frac{{\hat{\sigma }}_{SFE}^{2}}{n_{i}^{\prime }}+\frac{{\hat{\sigma }}_{TE}^{2}}{n_{o}^{\prime }}+\frac{{\hat{\sigma }}_{RRE}^{2}}{{n_{i}^{\prime }n}_{o}^{\prime }}}{{\hat{\sigma }}_{t}^{2}+\frac{{\hat{\sigma }}_{SFE}^{2}}{n_{i}^{\prime }}+\frac{{\hat{\sigma }}_{TE}^{2}}{n_{o}^{\prime }}+\frac{{\hat{\sigma }}_{RRE}^{2}}{{n_{i}^{\prime }n}_{o}^{\prime }}}$	$\frac{\frac{{\hat{\sigma }}_{SFE}^{2}}{n_{{\bar{H}}_{i}}^{\prime }}+\frac{{\hat{\sigma }}_{TE}^{2}}{n_{o}^{\prime }}+\frac{{\hat{\sigma }}_{RRE}^{2}}{n_{{\bar{H}}_{i}}^{\prime }n_{o}^{\prime }}}{{\hat{\sigma }}_{t}^{2}+{\hat{\sigma }}_{neg}^{2}+{\hat{\sigma }}_{pos}^{2}+\frac{{\hat{\sigma }}_{SFE}^{2}}{n_{{\bar{H}}_{i}}^{\prime }}+\frac{{\hat{\sigma }}_{TE}^{2}}{n_{o}^{\prime }}+\frac{{\hat{\sigma }}_{RRE}^{2}}{n_{{\bar{H}}_{i}}^{\prime }n_{o}^{\prime }}}$

Note. *t* = trait; *i* = items; *o* = occasion; SFE = specific-factor error; TE = transient error; RRE = random-response error; $n_{i}^{\prime }$ = the number of total items; $n_{neg}^{\prime }$ = the number of negatively phrased items; $n_{pos}^{\prime }$ = the number of positively phrased items; $n_{\bar{H}}^{\prime }$ = harmonic mean of $n_{neg}^{\prime }$ and $n_{pos}^{\prime }$; ${\hat{\sigma }}_{t}^{2}$ = trait variance; ${\hat{\sigma }}_{neg}^{2}$ = negative wording variance; ${\hat{\sigma }}_{pos}^{2}$ = positive wording variance; $\frac{{\hat{\sigma }}_{SFE}^{2}}{n_{i}^{\prime }}$ or $\frac{{\hat{\sigma }}_{SFE}^{2}}{n_{{\bar{H}}_{i}}^{\prime }}$ = specific-factor error variance; $\frac{{\hat{\sigma }}_{TE}^{2}}{n_{o}^{\prime }}$ = transient error variance; $\frac{{\hat{\sigma }}_{RRE}^{2}}{n_{i}^{\prime }n_{o}^{\prime }}$ or $\frac{{\hat{\sigma }}_{RRE}^{2}}{n_{{\bar{H}}_{i}}^{\prime }n_{o}^{\prime }}$ = random-response error variance. The harmonic mean for numbers of items across item wording parcels ($n_{{\bar{H}}_{i}}^{\prime }$) equals the number of parcels divided by the sum of the reciprocals of the number of items within each parcel. For example, if there are two parcels with four and six items, the harmonic mean would equal 2/(1/4 + 1/6) or 4.8. Primes are placed above the symbol *n* in the formulas to indicate that they can be altered for estimating the magnitude of indices when changing numbers of items and/or occasions. The variance components shown in the table are based on those defined in Table 1.

**Table 4:** Descriptive statistics and conventional reliability coefficients.

Index	Value
Occasion 1	
Mean: Total (Item)	52.08 (5.21)
SD: Total (Item)	12.71 (1.27)
Alpha	0.921
Omega	0.922
Occasion 2	
Mean: Total (Item)	52.46 (5.25)
SD: Total (Item)	12.98 (1.30)
Alpha	0.932
Omega	0.932
Test–retest	0.925

**Table 5:** Fit indices for one- and two-occasion designs.

	Index				
Design	Chi-Square	df	CFI	TLI	RMSEA
One occasion without wording	1406.849	35	0.829	0.780	0.148
One occasion with wording	297.139	25	0.966	0.939	0.078
Two occasions without wording	2490.847	170	0.897	0.885	0.087
Two occasions with wording	824.735	160	0.971	0.965	0.048

**Table 6:** Partitioning of SDQ-III Physical Appearance total score variance for one-occasion designs without and with item wording effects.

	Design/Proportion of Observed Score Variance	
Index	Without Wording	With Wording
Trait	0.921 (SE = 0.003; CI [0.915, 0.927])	0.856 (SE = 0.008; CI [0.839, 0.871])
Negative wording		0.044 (SE = 0.006; CI [0.034, 0.056])
Positive wording		0.043 (SE = 0.005; CI [0.034, 0.054])
Total wording		0.088 (SE = 0.007; CI [0.075, 0.102])
Trait plus total wording		0.943 (SE = 0.002; CI [0.938, 0.948])
Total error	0.079 (SE = 0.003; CI [0.073, 0.085])	0.057 (SE = 0.002; CI [0.052, 0.062])

Note. SE = standard error. Values within brackets represent Monte Carlo-based 95% confidence interval limits (MacKinnon et al., 2004; Preacher & Selig, 2012) obtained from the *semTools* package (Version 0.5-9) in R (Jorgensen et al., 2026). In the model with wording effects, trait proportion is equivalent to an omega hierarchical coefficient, and proportion of trait plus total wording is equivalent to an omega total coefficient. The sum for proportions for negative and positive wordings varies slightly from the proportion of total wording shown above due to rounding. Values reported here come directly from analyses reported in our Supplementary Materials.

**Table 7:** Single-occasion item-level partitioning for the SDQ-III Physical Appearance subscale.

	Effect/Proportion of Variance			
Item	Trait	Negative Wording	Positive Wording	Total Error
Item 1	0.706	0.000		0.293
Item 2	0.728	0.033		0.238
Item 3	0.300	0.317		0.383
Item 4	0.370	0.033		0.597
Item 5	0.466	0.274		0.260
Item 6	0.743		0.004	0.253
Item 7	0.431		0.400	0.168
Item 8	0.592		0.218	0.189
Item 9	0.574		0.008	0.419
Item 10	0.324		0.235	0.441

Note. For better readability, items 1–5 are grouped to represent negative wording, and items 6–10 are grouped to represent positive wording, but those items were not answered by respondents in that order. Within the SDQ-III, subscale content and item wording (negative and positive) are spiraled throughout the inventory. Actual item numbers are provided in our Supplementary Materials.

**Table 8:** Partitioning of SDQ-III Physical Appearance total score variance for two-occasion designs without and with item wording effects.

	Design/Proportion of Observed Score Variance	
Index	Without Wording	With Wording
Trait	0.876 (SE = 0.014; CI [0.845, 0.900])	0.817 (SE = 0.011, CI [0.794, 0.837])
Negative wording		0.046 (SE = 0.005, CI [0.037, 0.055])
Positive wording		0.040 (SE = 0.004; CI [0.032, 0.048])
Total wording		0.085 (SE = 0.006; CI [0.074, 0.099])
Trait plus total wording		0.902 (SE = 0.007, CI [0.888, 0.915])
Specific-factor error	0.034 (SE = 0.002; CI [0.031, 0.037])	0.022 (SE = 0.001; CI [0.020, 0.024])
Transient error	0.057 (SE = 0.013; CI [0.034, 0.087])	0.044 (SE = 0.006; CI [0.033, 0.057])
Random-response error	0.033 (SE = 0.001; CI [0.030, 0.036])	0.032 (SE = 0.001; CI [0.029, 0.035])
Total error	0.124 (SE = 0.014; CI [0.100, 0.155])	0.098 (SE = 0.007; CI [0.085, 0.112])

Note. SE = standard error. Values within brackets represent Monte Carlo-based 95% confidence interval limits (MacKinnon et al., 2004; Preacher & Selig, 2012) obtained from the *semTools* package in R (Jorgensen et al., 2026). In the model with wording effects, trait proportion is equivalent to an omega hierarchical coefficient, and proportion of trait plus total wording is equivalent to an omega total coefficient. The sum for proportions for negative and positive wordings varies slightly from the proportion of total wording shown above due to rounding. Values reported here come directly from analyses reported in our Supplementary Materials.

**Table 9:** Two-occasion item-level partitioning for the SDQ-III Physical Appearance subscale.

				Index			
Item	Trait	Negative Wording	Positive Wording	SFE	TE	RRE	Total Error
Item 1	0.683	0.002		0.098	0.041	0.176	0.315
Item 2	0.664	0.046		0.076	0.056	0.158	0.290
Item 3	0.293	0.305		0.149	0.016	0.237	0.402
Item 4	0.390	0.033		0.351	0.011	0.214	0.576
Item 5	0.439	0.275		0.050	0.027	0.208	0.285
Item 6	0.741		0.002	0.043	0.038	0.177	0.258
Item 7	0.424		0.357	0.027	0.029	0.164	0.220
Item 8	0.576		0.208	0.033	0.027	0.156	0.216
Item 9	0.581		0.006	0.201	0.024	0.188	0.413
Item 10	0.323		0.237	0.244	0.012	0.185	0.440

Note. SFE = specific-factor error; TE = transient error; RRE = random-response error. For better readability, items 1–5 are grouped to represent negative wording, and items 6–10 are grouped to represent positive wording, but those items were not answered by respondents in that order. Within the SDQ-III, subscale content and item wording (negative and positive) are spiraled throughout the inventory. Actual item numbers are provided in our Supplementary Materials.

## Data Availability

The dataset used in this study is not readily available due to Institutional Review Board restrictions. Inquiries about accessibility to the data should be forwarded to the lead author.

## Supplementary Materials

The following supporting information can be downloaded at https://www.mdpi.com/article/10.3390/bs16091636/s1, Supplementary Materials File S1: Instructional Online Supplement to Quantifying Effects of Item Wording and Multiple Sources of Measurement Error Using Extended Bifactor Models. Table S.1. Formulas for variance components within single and multiple occasion designs; Table S.2. Formulas for partitioning proportions of total score variance within single-occasion designs; Table S.3. Formulas for partitioning proportions of total score variance within multiple-occasion designs; Figure S3.1. Single-occasion design without wording effects; Figure S3.2. Single-occasion design with wording effects; Figure S4.1. Two-occasion design without wording effects; Figure S4.2. Two-occasion design with wording effects.
