# Supplementary material for: Quantifying Effects of Item Wording and Multiple Sources of Measurement Error Using Extended Bifactor Models
Source: Behav Sci (Basel). 2026 Sep 13;16(9):1636. doi: 10.3390/bs16091636 (PMC13603944; doi:10.3390/bs16091636)
Supplement: Supplementary file 1 [file behavsci-16-01636-s001.zip › behavsci-4337334-supplementary.pdf]

Instructional Online Supplement to

"Quantifying Effects of Item Wording and Multiple Sources of Measurement Error using Extended Bifactor Models"

By Walter P. Vispoel, Hyeryung Lee, and Tingting Chen

Contents

1. Data Preparation 2
2. Formulas for Analyzed Designs 3
3. Single-Occasion Designs 6
   3.1 Single-occasion design without wording effects 6
   3.2 Single-occasion design with wording effects 7
4. Two-Occasion designs 11
   4.1 Two-occasion design without wording effects 11
   4.2 Two-occasion design with wording effects 14
Abbreviations used throughout the supplement 20
References 21

1. Data Preparation

Examples used in this supplement represent one- and two-occasion code and results for Physical Appearance subscale scores from the Self-Description Questionnaire-III (SDQ-III; Marsh, 1992). Following essential data cleaning, the dataset, as well as relevant R libraries, are loaded. All item variables are renamed here with the labels 'T1_' for Occasion 1 and 'T2_' for Occasion 2. When using the *lavaan* (Rosseel, 2012; Rosseel et al., 2025) and *semTools* (Jorgensen et al., 2025) packages in *R* to perform confirmatory factor analyses, data must be arranged in a wide format that has a column for each variable. Sample code is provided below for using these packages to estimate proportions of total score variance accounted for the trait of interest, item wording, and sources of measurement error with corresponding 95% Monte Carlo confidence intervals provided. The item numbers following the notation "c(" are those used in the present dataset for the Physical Appearance subscale. Adjustments would need to be made when using other datasets.

```
library(lavaan)
library(semTools)

SDQ3 <- read.csv('SDQ3.csv')

SDQ3_appear <- SDQ3[,c(paste0("T1S",c(12, 25, 38, 51, 64, 77, 90, 103, 116, 129)),
                       paste0("T2S",c(12, 25, 38, 51, 64, 77, 90, 103, 116, 129)))]
```

In sections to follow, we provide code and output for a single-occasion design without wording effects, a single-occasion design with wording effects, a two-occasion design without wording effects, and two-occasion design with wording effects. Item-level partitioning results are also provided for the designs that included wording effects. Within Section 2, tables from the main article are reproduced with formulas for estimating variance components and proportions of observed score variance accounted for by the trait of interest, item wording, and associated sources of measurement error. The relevant figure for each structural equation model from the main article also precedes the code and output for that design. All analyses are based on responses to the Physical Appearance subscale from the SDQ-III. The output generated by R or other statistical packages should always by checked for issues of non-convergence and counterintuitive results for any analyzed design.

2. Formulas for Analyzed Designs

Table S.1. Formulas for variance components within single and multiple occasion designs.

Design / Index	Without Wording Effects	With Wording Effects
Single Occasion		
Trait VC	$\hat{\sigma}_t^2 = \left(\frac{\sum_{i=1}^{n_i} \hat{\delta}_i}{n_i}\right)^2 \hat{V}_t$	$\hat{\sigma}_t^2 = \left(\frac{\sum_{i=1}^{n_i} \hat{\delta}_i}{n_{\bar{H}_i}}\right)^2 \hat{V}_t$
Negative Item Wording VC		$\hat{\sigma}_{neg}^2 = \left(\frac{\sum_{j=1}^{n_{neg}} \hat{\lambda}_i}{n_{neg}}\right)^2 \hat{V}_{neg}$
Positive Item Wording VC		$\hat{\sigma}_{pos}^2 = \left(\frac{\sum_{k=1}^{n_{pos}} \hat{\lambda}_i}{n_{pos}}\right)^2 \hat{V}_{pos}$
Total Measurement Error VC	$\hat{\sigma}_{error}^2 = \frac{\sum_{i=1}^{n_i} \hat{U}_i}{n_i}$	$\hat{\sigma}_{error}^2 = \frac{\sum_{i=1}^{n_i} \hat{U}_i}{n_{\bar{H}_i}}$
Multiple Occasions		
Trait VC	$\hat{\sigma}_t^2 = \left(\frac{\sum_{i=1}^{n_i} \hat{\delta}_i}{n_i}\right)^2 \hat{V}_t$	$\hat{\sigma}_t^2 = \left(\frac{\sum_{i=1}^{n_i} \hat{\delta}_i}{n_{\bar{H}_i}}\right)^2 \hat{V}_t$
Negative Item Wording VC		$\hat{\sigma}_{neg}^2 = \left(\frac{\sum_{j=1}^{n_{neg}} \hat{\lambda}_j}{n_{neg}}\right)^2 \hat{V}_{neg}$
Positive Item Wording VC		$\hat{\sigma}_{pos}^2 = \left(\frac{\sum_{k=1}^{n_{pos}} \hat{\lambda}_k}{n_{pos}}\right)^2 \hat{V}_{pos}$
Transient Error VC	$\hat{\sigma}_{TE}^2 = \left(\frac{\sum_{i=1}^{n_i} \hat{\beta}_i}{n_i}\right)^2 \hat{V}_{TE}$	$\hat{\sigma}_{TE}^2 = \left(\frac{\sum_{i=1}^{n_i} \hat{\beta}_i}{n_{\bar{H}_i}}\right)^2 \hat{V}_{TE}$
Specific-Factor Error VC	$\hat{\sigma}_{SFE}^2 = \frac{\sum_{i=1}^{n_i}(\hat{\gamma}_i{}^2\, \hat{V}_{SFE})}{n_i}$	$\hat{\sigma}_{SFE}^2 = \frac{\sum_{i=1}^{n_i}(\hat{\gamma}_i{}^2\, \hat{V}_{SFE})}{n_{\bar{H}_i}}$
Random-Response Error VC	$\hat{\sigma}_{RRE}^2 = \frac{\sum_{i=1}^{n_i} \hat{U}_i}{n_i}$	$\hat{\sigma}_{RRE}^2 = \frac{\sum_{i=1}^{n_i} \hat{U}_i}{n_{\bar{H}_i}}$

Note. t = trait; i = items; j = negatively phrased items; k = positively phrased items; o = occasions; VC = variance component; SFE = specific-factor error; TE = transient error; RRE = random-response error; n_i = number of total items; n_{neg} = number of negatively phrased items; n_{pos} = number of positively phrased items; $n_{\bar{H}_i}$ = harmonic mean of n_{neg} and n_{pos}; $\hat{V}$ = factor variance; $\hat{\delta}$ = estimated factor loading for the trait factor; $\hat{\lambda}_{neg}$= estimated factor loading for the negative item wording factor; $\hat{\lambda}_{pos}$ = estimated factor loading for the positive item wording factor; $\hat{\gamma}$ = estimated factor loading for an item factor; $\hat{\beta}$ = estimated factor loading for an occasion factor. The harmonic mean for numbers of items across item wording parcels ($n'_{\bar{H}_i}$) equals the number of parcels divided by the sum of the reciprocals of the number of items within each parcel. For example, if there are two parcels with four and six items, the harmonic mean would equal 2/(1/4 + 1/6) or 4.8. For the present factor model designs, $\hat{V}_t$, $\hat{V}_{neg}$, $\hat{V}_{pos}$, $\hat{V}_{SFE}$ and $\hat{V}_{TE}$ are set equal to 1. The formulas presented for conditions with wording effects assume an equal number of positively and negatively phrased items. If the number of items in each category differs, each component (e.g., loading, variance, or uniqueness) should be proportionally adjusted based on the relative number of positively and negatively phrased items in the total set.

Table S.2. Formulas for partitioning proportions of total score variance within single-occasion designs.

Index/Design	Formula	
	Without Item Wording	With Item Wording
$\hat{\omega}$ total coefficient	$\frac{\hat{\sigma}_t^2}{\hat{\sigma}_t^2 + \frac{\hat{\sigma}^2_{total\,error}}{n'_i}}$	$\frac{\hat{\sigma}_t^2 + \hat{\sigma}^2_{neg} + \hat{\sigma}^2_{pos}}{\hat{\sigma}_t^2 + \hat{\sigma}^2_{neg} + \hat{\sigma}^2_{pos} + \frac{\hat{\sigma}^2_{total\,error}}{n'_{\bar{H}_i}}}$
$\hat{\omega}$ hierarchical coefficient		$\frac{\hat{\sigma}_t^2}{\hat{\sigma}_t^2 + \hat{\sigma}^2_{neg} + \hat{\sigma}^2_{pos} + \frac{\hat{\sigma}^2_{total\,error}}{n'_{\bar{H}_i}}}$
Negative wording proportion		$\frac{\hat{\sigma}^2_{neg}}{\hat{\sigma}_t^2 + \hat{\sigma}^2_{neg} + \hat{\sigma}^2_{pos} + \frac{\hat{\sigma}^2_{total\,error}}{n'_{\bar{H}_i}}}$
Positive wording proportion		$\frac{\hat{\sigma}^2_{pos}}{\hat{\sigma}_t^2 + \hat{\sigma}^2_{neg} + \hat{\sigma}^2_{pos} + \frac{\hat{\sigma}^2_{total\,error}}{n'_{\bar{H}_i}}}$
Total wording proportion		$\frac{\hat{\sigma}^2_{neg} + \hat{\sigma}^2_{pos}}{\hat{\sigma}_t^2 + \hat{\sigma}^2_{neg} + \hat{\sigma}^2_{pos} + \frac{\hat{\sigma}^2_{total\,error}}{n'_{\bar{H}_i}}}$
Total error proportion	$\frac{\frac{\hat{\sigma}^2_{total\,error}}{n'_i}}{\hat{\sigma}_t^2 + \frac{\hat{\sigma}^2_{total\,error}}{n'_i}}$	$\frac{\frac{\hat{\sigma}^2_{total\,error}}{n'_{\bar{H}_i}}}{\hat{\sigma}_t^2 + \hat{\sigma}^2_{neg} + \hat{\sigma}^2_{pos} + \frac{\hat{\sigma}^2_{total\,error}}{n'_{\bar{H}_i}}}$

Note. t = trait; i = items; o = occasion; n'_{neg} = the number of negatively phrased items; n'_{pos} = the number of positively phrased items; n'_i = the number of total items; $n'_{\bar{H}}$ = harmonic mean of n'_{neg} and n'_{pos}. In the single-occasion designs: $\hat{\sigma}_t^2$ = trait variance; $\hat{\sigma}^2_{neg}$ = negative wording variance; $\hat{\sigma}^2_{pos}$ = positive wording variance; $\frac{\hat{\sigma}^2_{total\,error}}{n'_i}$ or $\frac{\hat{\sigma}^2_{total\,error}}{n'_{\bar{H}_i}}$ = total error variance. The harmonic mean for numbers of items across item wording parcels ($n'_{\bar{H}_i}$) equals the number of parcels divided by the sum of the reciprocals of the number of items within each parcel. For example, if there are two parcels with four and six items, the harmonic mean would equal 2/(1/4 + 1/6) or 4.8. Primes are placed above the symbol n in the formulas to indicate that they can be altered for estimating the magnitude of indices when changing numbers of items sharing characteristics of those originally analyzed. The variance components shown in the table are based on those defined in Table S-1.

Table S.3. Formulas for partitioning proportions of total score variance within multiple-occasion designs.

Index/Design	Formula	
	Without Item Wording	With Item Wording
$\hat{\omega}$ total coefficient	$\frac{\hat{\sigma}_t^2}{\hat{\sigma}_t^2 + \frac{\hat{\sigma}_{SFE}^2}{n_i'} + \frac{\hat{\sigma}_{TE}^2}{n_o'} + \frac{\hat{\sigma}_{RRE}^2}{n_i'n_o'}}$	$\frac{\hat{\sigma}_t^2 + \hat{\sigma}_{neg}^2 + \hat{\sigma}_{pos}^2}{\hat{\sigma}_t^2 + \hat{\sigma}_{neg}^2 + \hat{\sigma}_{pos}^2 + \frac{\hat{\sigma}_{SFE}^2}{n_{\bar{H}_i}'} + \frac{\hat{\sigma}_{TE}^2}{n_o'} + \frac{\hat{\sigma}_{RRE}^2}{n_{\bar{H}_i}'n_o'}}$
$\hat{\omega}$ hierarchical coefficient		$\frac{\hat{\sigma}_t^2}{\hat{\sigma}_t^2 + \hat{\sigma}_{neg}^2 + \hat{\sigma}_{pos}^2 + \frac{\hat{\sigma}_{SFE}^2}{n_{\bar{H}_i}'} + \frac{\hat{\sigma}_{TE}^2}{n_o'} + \frac{\hat{\sigma}_{RRE}^2}{n_{\bar{H}_i}'n_o'}}$
Negative wording proportion		$\frac{\hat{\sigma}_{neg}^2}{\hat{\sigma}_t^2 + \hat{\sigma}_{neg}^2 + \hat{\sigma}_{pos}^2 + \frac{\hat{\sigma}_{SFE}^2}{n_{\bar{H}_i}'} + \frac{\hat{\sigma}_{TE}^2}{n_o'} + \frac{\hat{\sigma}_{RRE}^2}{n_{\bar{H}_i}'n_o'}}$
Positive wording proportion		$\frac{\hat{\sigma}_{pos}^2}{\hat{\sigma}_t^2 + \hat{\sigma}_{neg}^2 + \hat{\sigma}_{pos}^2 + \frac{\hat{\sigma}_{SFE}^2}{n_{\bar{H}_i}'} + \frac{\hat{\sigma}_{TE}^2}{n_o'} + \frac{\hat{\sigma}_{RRE}^2}{n_{\bar{H}_i}'n_o'}}$
Total wording proportion		$\frac{\hat{\sigma}_{neg}^2 + \hat{\sigma}_{pos}^2}{\hat{\sigma}_t^2 + \hat{\sigma}_{neg}^2 + \hat{\sigma}_{pos}^2 + \frac{\hat{\sigma}_{SFE}^2}{n_{\bar{H}_i}'} + \frac{\hat{\sigma}_{TE}^2}{n_o'} + \frac{\hat{\sigma}_{RRE}^2}{n_{\bar{H}_i}'n_o'}}$
Specific-factor error proportion	$\frac{\frac{\hat{\sigma}_{SFE}^2}{n_i'}}{\hat{\sigma}_t^2 + \frac{\hat{\sigma}_{SFE}^2}{n_i'} + \frac{\hat{\sigma}_{TE}^2}{n_o'} + \frac{\hat{\sigma}_{RRE}^2}{n_i'n_o'}}$	$\frac{\frac{\hat{\sigma}_{SFE}^2}{n_{\bar{H}_i}'}}{\hat{\sigma}_t^2 + \hat{\sigma}_{neg}^2 + \hat{\sigma}_{pos}^2 + \frac{\hat{\sigma}_{SFE}^2}{n_{\bar{H}_i}'} + \frac{\hat{\sigma}_{TE}^2}{n_o'} + \frac{\hat{\sigma}_{RRE}^2}{n_{\bar{H}_i}'n_o'}}$
Transient error proportion	$\frac{\frac{\hat{\sigma}_{TE}^2}{n_o'}}{\hat{\sigma}_t^2 + \frac{\hat{\sigma}_{SFE}^2}{n_i'} + \frac{\hat{\sigma}_{TE}^2}{n_o'} + \frac{\hat{\sigma}_{RRE}^2}{n_i'n_o'}}$	$\frac{\frac{\hat{\sigma}_{TE}^2}{n_o'}}{\hat{\sigma}_t^2 + \hat{\sigma}_{neg}^2 + \hat{\sigma}_{pos}^2 + \frac{\hat{\sigma}_{SFE}^2}{n_{\bar{H}_i}'} + \frac{\hat{\sigma}_{TE}^2}{n_o'} + \frac{\hat{\sigma}_{RRE}^2}{n_{\bar{H}_i}'n_o'}}$
Random-response error proportion	$\frac{\frac{\hat{\sigma}_{RRE}^2}{n_i'n_o'}}{\hat{\sigma}_t^2 + \frac{\hat{\sigma}_{SFE}^2}{n_i'} + \frac{\hat{\sigma}_{TE}^2}{n_o'} + \frac{\hat{\sigma}_{RRE}^2}{n_i'n_o'}}$	$\frac{\frac{\hat{\sigma}_{RRE}^2}{n_{\bar{H}_i}'n_o'}}{\hat{\sigma}_t^2 + \hat{\sigma}_{neg}^2 + \hat{\sigma}_{pos}^2 + \frac{\hat{\sigma}_{SFE}^2}{n_{\bar{H}_i}'} + \frac{\hat{\sigma}_{TE}^2}{n_o'} + \frac{\hat{\sigma}_{RRE}^2}{n_{\bar{H}_i}'n_o'}}$
Total error proportion	$\frac{\frac{\hat{\sigma}_{SFE}^2}{n_i'} + \frac{\hat{\sigma}_{TE}^2}{n_o'} + \frac{\hat{\sigma}_{RRE}^2}{n_i'n_o'}}{\hat{\sigma}_t^2 + \frac{\hat{\sigma}_{SFE}^2}{n_i'} + \frac{\hat{\sigma}_{TE}^2}{n_o'} + \frac{\hat{\sigma}_{RRE}^2}{n_i'n_o'}}$	$\frac{\frac{\hat{\sigma}_{SFE}^2}{n_{\bar{H}_i}'} + \frac{\hat{\sigma}_{TE}^2}{n_o'} + \frac{\hat{\sigma}_{RRE}^2}{n_{\bar{H}_i}'n_o'}}{\hat{\sigma}_t^2 + \hat{\sigma}_{neg}^2 + \hat{\sigma}_{pos}^2 + \frac{\hat{\sigma}_{SFE}^2}{n_{\bar{H}_i}'} + \frac{\hat{\sigma}_{TE}^2}{n_o'} + \frac{\hat{\sigma}_{RRE}^2}{n_{\bar{H}_i}'n_o'}}$

Note. t = trait; i = items; o = occasion; SFE = specific-factor error; TE = transient error; RRE = random-response error; n_i' = the number of total items; n_{neg}' = the number of negatively phrased items; n_{pos}' = the number of positively phrased items; $n_{\bar{H}}'$ = harmonic mean of n_{neg}' and n_{pos}'. $\hat{\sigma}_t^2$ = trait variance; $\hat{\sigma}_{neg}^2$ = negative wording variance; $\hat{\sigma}_{pos}^2$ = positive wording variance; $\frac{\hat{\sigma}_{SFE}^2}{n_i'}$ or $\frac{\hat{\sigma}_{SFE}^2}{n_{\bar{H}_i}'}$ = specific-factor error variance; $\frac{\hat{\sigma}_{TE}^2}{n_o'}$ =transient error variance; $\frac{\hat{\sigma}_{RRE}^2}{n_i'n_o'}$ or $\frac{\hat{\sigma}_{RRE}^2}{n_{\bar{H}_i}'n_o'}$= random-response error variance. The harmonic mean for numbers of items across item wording parcels ($n_{\bar{H}_i}'$) equals the number of parcels divided by the sum of the reciprocals of the number of items within each parcel. For example, if there are two parcels with four and six items, the harmonic mean would equal 2/(1/4 + 1/6) or 4.8. Primes are placed above the symbol n in the formulas to indicate that they can be altered for estimating the magnitude of indices when changing numbers of items and/or occasions sharing characteristics of those originally analyzed. The variance components shown in the table are based on those defined in Table S-1.

3. Single-Occasion Designs

3.1 Single-occasion design without wording effects

Figure S3.1. Single-occasion design without wording effects.

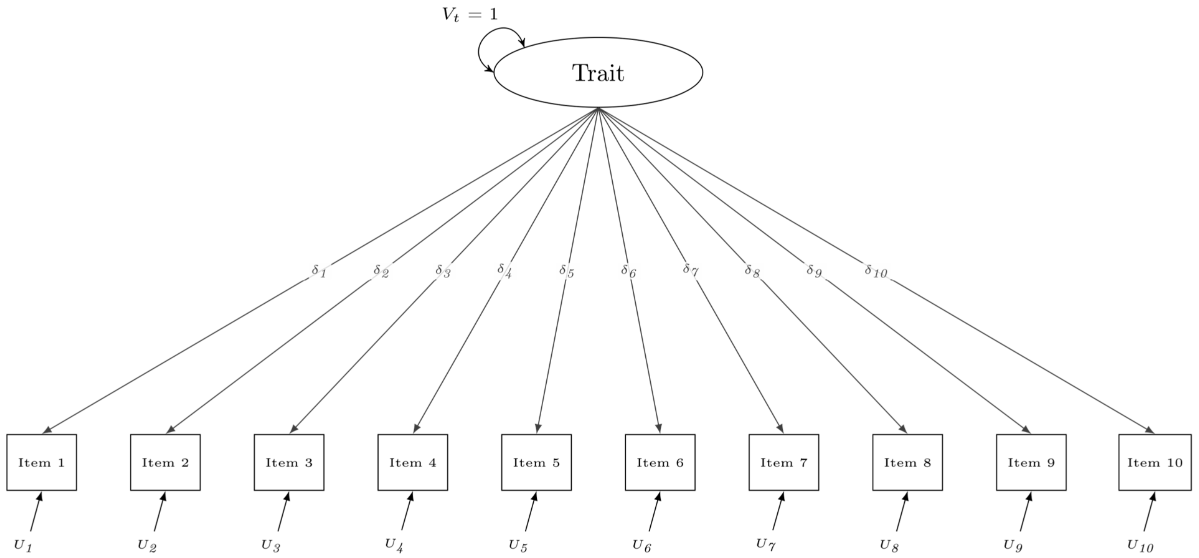


```
Model <- 'Person =~ NA*T1S12 + dlt1*T1S12 + dlt2*T1S25 + dlt3*T1S38 + dlt4*T1
S51 + dlt5*T1S64 + dlt6*T1S77+ dlt7*T1S90 + dlt8*T1S103 + dlt9*T1S116 + dlt10
*T1S129

## person variance
  Person ~~ 1*Person

  # 12, 25, 38, 51, 64, 77, 90, 103, 116, 129
## p x i interaction variance (+ error)
  T1S12 ~~ U_pi1*T1S12
  T1S25 ~~ U_pi2*T1S25
  T1S38 ~~ U_pi3*T1S38
  T1S51 ~~ U_pi4*T1S51
  T1S64 ~~ U_pi5*T1S64
  T1S77 ~~ U_pi6*T1S77
  T1S90 ~~ U_pi7*T1S90
  T1S103 ~~ U_pi8*T1S103
  T1S116 ~~ U_pi9*T1S116
  T1S129 ~~ U_pi10*T1S129

Trait_VC := ((dlt1 + dlt2+ dlt3+dlt4+ dlt5+ dlt6+ dlt7+ dlt8+ dlt9+ dlt10)/10
)^2
Error_VC := (U_pi1+U_pi2+U_pi3+U_pi4+U_pi5+U_pi6+U_pi7+U_pi8+U_pi9+U_pi10)/10
Sum_VCs:=Trait_VC + Error_VC/10
```

```
Omega_total_coef := Trait_VC /Sum_VCs
Total_error_prop:= (Error_VC/10)/Sum_VCs'


Results <- lavaan(Model, data = SDQ3_appear,estimator = "MLR", orthogonal = T
RUE)
fitmeasures(Results)[c("chisq.scaled","df.scaled","cfi.scaled","tli.scaled","
rmsea.scaled")]

## chisq.scaled    df.scaled   cfi.scaled   tli.scaled rmsea.scaled
## 1406.8485211   35.0000000    0.8288245    0.7799172    0.1477290

mc_ci <- monteCarloCI(Results,level = 0.95)
pe <- parameterEstimates(Results)
se <- pe[pe$op == ":=", "se"]
round(cbind(mc_ci, se),3)

##                    est ci.lower ci.upper    se
## Trait_VC         1.480    1.372    1.594 0.056
## Error_VC         1.267    1.218    1.316 0.025
## Sum_VCs          1.606    1.500    1.721 0.056
## Omega_total_coef 0.921    0.915    0.927 0.003
## Total_error_prop 0.079    0.073    0.085 0.003
```

3.2 Single-occasion design with wording effects

Figure S3.2. Single-occasion design with wording effects.

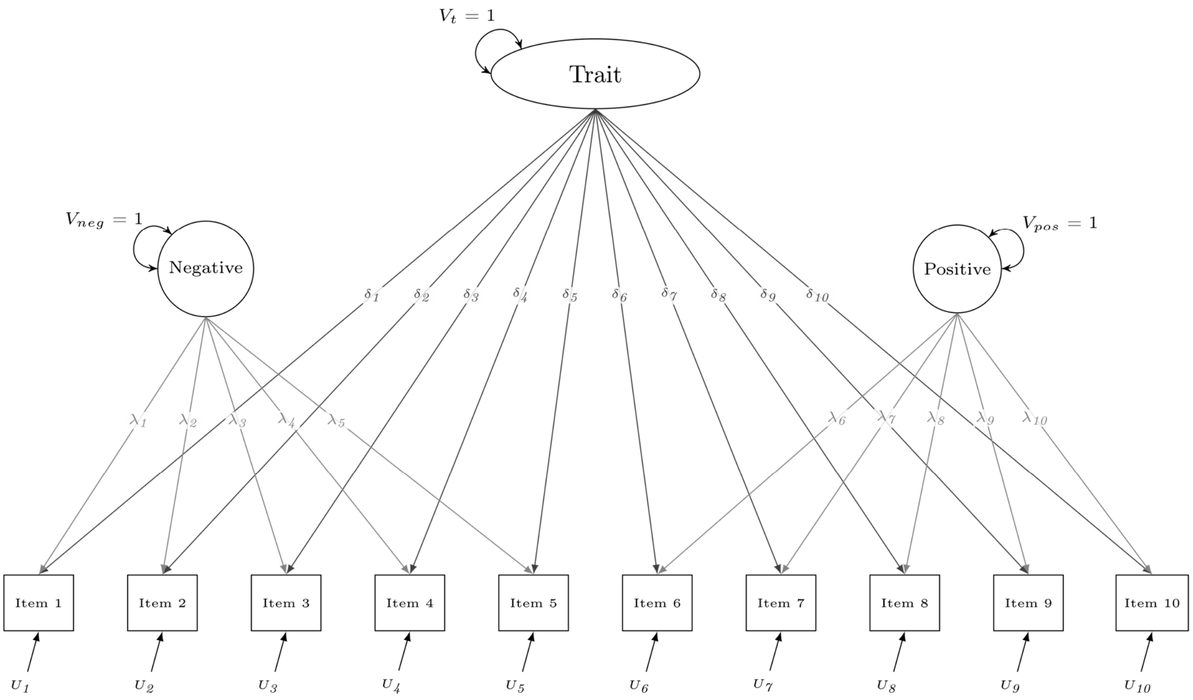

```
Model  <- ' Person =~ NA*T1S12 + dlt1*T1S12 + dlt2*T1S25 + dlt3*T1S38 + dlt4*
T1S51 + dlt5*T1S64 + dlt6*T1S77+ dlt7*T1S90 + dlt8*T1S103 + dlt9*T1S116 + dlt
10*T1S129

## person variance
  Person ~~ 1*Person

NEG =~ NA*T1S12+lmd1*T1S12 + lmd2*T1S38 + lmd3*T1S64 + lmd4*T1S90 + lmd5*T1S1
16
POS =~ NA*T1S25+lmd6*T1S25 + lmd7*T1S51 + lmd8*T1S77 + lmd9*T1S103 + lmd10*T1
S129

NEG ~~ 1*NEG
POS ~~ 1*POS

  # 12, 25, 38, 51, 64, 77, 90, 103, 116, 129
## p x i interaction variance (+ error)
  T1S12 ~~ U_pi1*T1S12
  T1S25 ~~ U_pi2*T1S25
  T1S38 ~~ U_pi3*T1S38
  T1S51 ~~ U_pi4*T1S51
  T1S64 ~~ U_pi5*T1S64
  T1S77 ~~ U_pi6*T1S77
  T1S90 ~~ U_pi7*T1S90
  T1S103 ~~ U_pi8*T1S103
  T1S116 ~~ U_pi9*T1S116
  T1S129 ~~ U_pi10*T1S129


Trait_VC := ((dlt1 + dlt2+ dlt3+dlt4+ dlt5+ dlt6+ dlt7+ dlt8+ dlt9+ dlt10)/5)
^2
Neg_VC := ((lmd1+lmd2+lmd3+lmd4+lmd5)/5)^2
Pos_VC := ((lmd6+lmd7+lmd8+lmd9+lmd10)/5)^2
Error_VC := (U_pi1+U_pi2+U_pi3+U_pi4+U_pi5+U_pi6+U_pi7+U_pi8+U_pi9+U_pi10)/5
Sum_VCs:=Trait_VC + Error_VC/5 + Neg_VC + Pos_VC
Omega_total_coef := (Trait_VC + Neg_VC + Pos_VC) /Sum_VCs
Omega_hierarchical_coef := Trait_VC/Sum_VCs
NEG_prop := Neg_VC/Sum_VCs
POS_prop := Pos_VC/Sum_VCs
Total_wording_prop := NEG_prop + POS_prop
Total_error_prop:= (Error_VC/5)/Sum_VCs
'
make_item_code <- function(i) {
  item <- c(1, 3, 5, 7, 9, 2, 4, 6, 8, 10)[i]

  if (i <= 5) {
    k <- i
    wording_par <- paste0("lmd", k)
    label <- "Neg"
```

```
  } else {
    k <- i
    wording_par <- paste0("lmd", k)
    label <- "Pos"
  }

  c(
    paste0("sum_", i, " := dlt", item, "^2 + lmd",i, "^2 + U_pi", item),
    paste0("Trait_", i, " := dlt", item, "^2/sum_", i),
    paste0(label, "_", i, " := lmd",i, "^2/sum_", i),
    paste0("Error_", i, " := U_pi", item, "/sum_", i)
  )
}

item_level <- paste(unlist(lapply(1:10, make_item_code)), collapse = "\n")


Results <- lavaan(paste(Model, item_level), data = SDQ3_appear,estimator = "M
LR",orthogonal = TRUE)
fitmeasures(Results)[c("chisq.scaled","df.scaled","cfi.scaled","tli.scaled","
rmsea.scaled")]

## chisq.scaled    df.scaled   cfi.scaled   tli.scaled rmsea.scaled
## 297.13864794  25.00000000   0.96604328   0.93887790   0.07785239

mc_ci <- monteCarloCI(Results,level = 0.95)
pe <- parameterEstimates(Results)
se <- pe[pe$op == ":=", "se"]
round(cbind(mc_ci, se),3)

##                             est ci.lower ci.upper    se
## Trait_VC                  5.412    4.972    5.881 0.231
## Neg_VC                    0.280    0.217    0.351 0.034
## Pos_VC                    0.274    0.216    0.338 0.031
## Error_VC                  1.798    1.717    1.879 0.041
## Sum_VCs                   6.326    5.902    6.776 0.224
## Omega_total_coef          0.943    0.938    0.948 0.002
## Omega_hierarchical_coef 0.856    0.839    0.871 0.008
## NEG_prop                  0.044    0.034    0.056 0.006
## POS_prop                  0.043    0.034    0.054 0.005
## Total_wording_prop        0.088    0.075    0.102 0.007
## Total_error_prop          0.057    0.052    0.062 0.002
## sum_1                     2.076    1.907    2.263 0.091
## Trait_1                   0.706    0.650    0.756 0.027
## Neg_1                     0.000    0.000    0.010 0.001
## Error_1                   0.293    0.241    0.345 0.026
## sum_2                     2.672    2.495    2.869 0.095
## Trait_2                   0.728    0.681    0.771 0.023
## Neg_2                     0.033    0.012    0.065 0.013
## Error_2                   0.238    0.208    0.269 0.015
```

```
## sum_3                        3.773    3.600    3.978 0.095
## Trait_3                      0.300    0.257    0.344 0.022
## Neg_3                        0.317    0.211    0.434 0.057
## Error_3                      0.383    0.266    0.495 0.058
## sum_4                        2.360    2.221    2.516 0.075
## Trait_4                      0.370    0.327    0.414 0.022
## Neg_4                        0.033    0.014    0.059 0.012
## Error_4                      0.597    0.553    0.638 0.022
## sum_5                        3.348    3.190    3.540 0.088
## Trait_5                      0.466    0.410    0.516 0.027
## Neg_5                        0.274    0.163    0.405 0.062
## Error_5                      0.260    0.163    0.348 0.047
## sum_6                        1.845    1.703    1.995 0.075
## Trait_6                      0.743    0.690    0.794 0.027
## Pos_6                        0.004    0.000    0.015 0.004
## Error_6                      0.253    0.196    0.309 0.029
## sum_7                        2.959    2.791    3.140 0.088
## Trait_7                      0.431    0.379    0.484 0.027
## Pos_7                        0.400    0.335    0.468 0.034
## Error_7                      0.168    0.122    0.215 0.024
## sum_8                        2.827    2.655    3.010 0.091
## Trait_8                      0.592    0.540    0.643 0.027
## Pos_8                        0.218    0.172    0.269 0.025
## Error_8                      0.189    0.157    0.221 0.016
## sum_9                        2.002    1.844    2.175 0.084
## Trait_9                      0.574    0.527    0.619 0.023
## Pos_9                        0.008    0.001    0.022 0.006
## Error_9                      0.419    0.370    0.467 0.025
## sum_10                       3.817    3.619    4.029 0.105
## Trait_10                     0.324    0.281    0.368 0.022
## Pos_10                       0.235    0.193    0.279 0.022
## Error_10                     0.441    0.398    0.485 0.022
```

4. Two-Occasion designs

4.1 Two-occasion design without wording effects

Figure S4.1. Two-occasion design without wording effects.

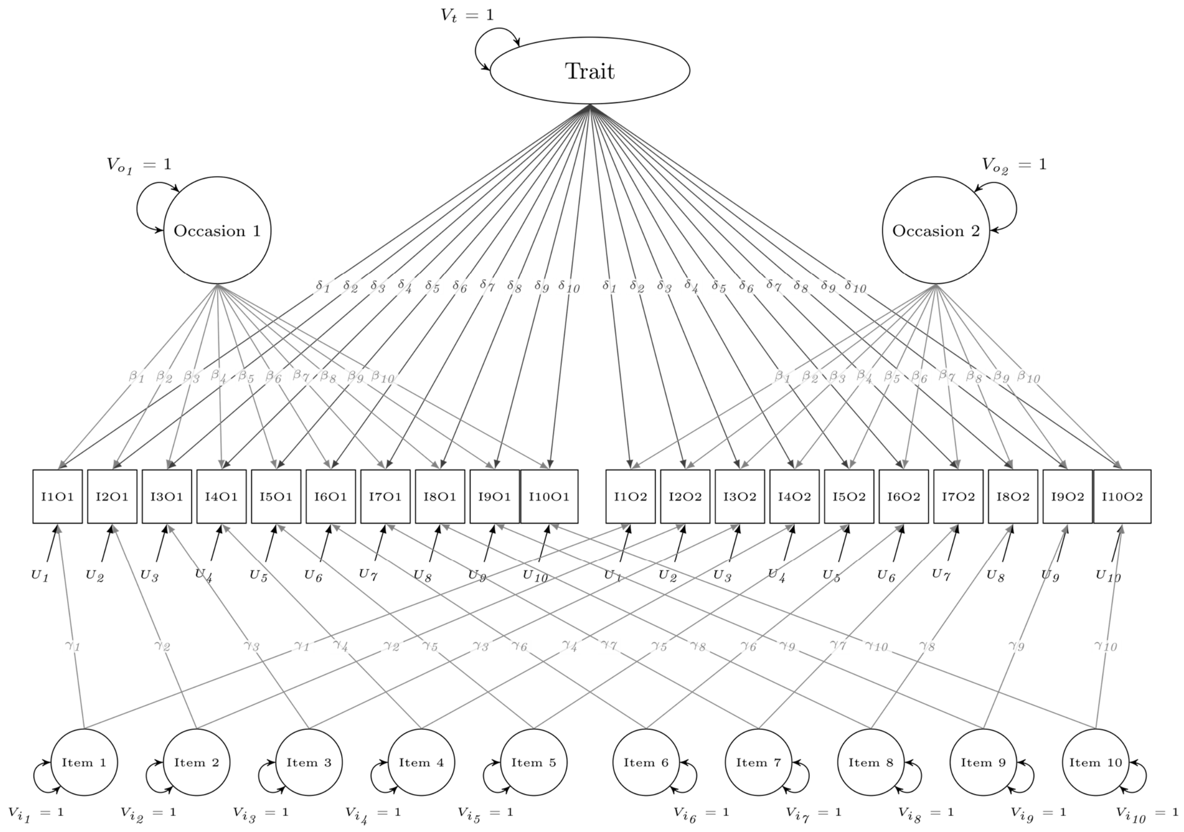


```
Model <- 'Person =~ NA*T1S12 + dlt1*T1S12 + dlt2*T1S25 + dlt3*T1S38 + dlt4*T1
S51 + dlt5*T1S64 + dlt6*T1S77+ dlt7*T1S90 + dlt8*T1S103 + dlt9*T1S116 + dlt10
*T1S129+ dlt1*T2S12 + dlt2*T2S25 + dlt3*T2S38 + dlt4*T2S51 + dlt5*T2S64 + dlt
6*T2S77 + dlt7*T2S90 + dlt8*T2S103 + dlt9*T2S116 + dlt10*T2S129
  Item1 =~ NA*T1S12 + gma1*T1S12 + gma1*T2S12
  Item2 =~ NA*T1S25 + gma2*T1S25 + gma2*T2S25
  Item3 =~ NA*T1S38 + gma3*T1S38 + gma3*T2S38
  Item4 =~ NA*T1S51 + gma4*T1S51 + gma4*T2S51
  Item5 =~ NA*T1S64 + gma5*T1S64 + gma5*T2S64
  Item6 =~ NA*T1S77 + gma6*T1S77 + gma6*T2S77
  Item7 =~ NA*T1S90 + gma7*T1S90 + gma7*T2S90
  Item8 =~ NA*T1S103 + gma8*T1S103 + gma8*T2S103
  Item9 =~ NA*T1S116 + gma9*T1S116 + gma9*T2S116
  Item10 =~ NA*T1S129 + gma10*T1S129 + gma10*T2S129
```

```
Occasion1 =~ NA*T1S12 + bta1*T1S12 + bta2*T1S25 + bta3*T1S38 + bta4*T1S51 + b
ta5*T1S64 + bta6*T1S77 + bta7*T1S90 + bta8*T1S103 + bta9*T1S116 + bta10*T1S12
9
Occasion2 =~ NA*T2S12 + bta1*T2S12 + bta2*T2S25 + bta3*T2S38 + bta4*T2S51 + b
ta5*T2S64 + bta6*T2S77 + bta7*T2S90 + bta8*T2S103 + bta9*T2S116 + bta10*T2S12
9

## person variance
  Person ~~ 1*Person

## p x i interaction variance
  Item1 ~~ 1*Item1
  Item2 ~~ 1*Item2
  Item3 ~~ 1*Item3
  Item4 ~~ 1*Item4
  Item5 ~~ 1*Item5
  Item6 ~~ 1*Item6
  Item7 ~~ 1*Item7
  Item8 ~~ 1*Item8
  Item9 ~~ 1*Item9
  Item10 ~~ 1*Item10
## p x o interaction variance
  Occasion1 ~~ 1*Occasion1
  Occasion2 ~~ 1*Occasion2

  # 12, 25, 38, 51, 64, 77, 90, 103, 116, 129
## p x i x o interaction variance (+ error)
  T1S12 ~~ U_pio1*T1S12
  T1S25 ~~ U_pio2*T1S25
  T1S38 ~~ U_pio3*T1S38
  T1S51 ~~ U_pio4*T1S51
  T1S64 ~~ U_pio5*T1S64
  T1S77 ~~ U_pio6*T1S77
  T1S90 ~~ U_pio7*T1S90
  T1S103 ~~ U_pio8*T1S103
  T1S116 ~~ U_pio9*T1S116
  T1S129 ~~ U_pio10*T1S129

  T2S12 ~~ U_pio1*T2S12
  T2S25 ~~ U_pio2*T2S25
  T2S38 ~~ U_pio3*T2S38
  T2S51 ~~ U_pio4*T2S51
  T2S64 ~~ U_pio5*T2S64
  T2S77 ~~ U_pio6*T2S77
  T2S90 ~~ U_pio7*T2S90
  T2S103 ~~ U_pio8*T2S103
  T2S116 ~~ U_pio9*T2S116
  T2S129 ~~ U_pio10*T2S129
```

```
Trait_VC := ((dlt1+dlt2+dlt3+dlt4+dlt5+dlt6+dlt7+dlt8+dlt9+dlt10)/10)^2
SFE_VC :=(gma1^2+gma2^2+gma3^2+gma4^2+gma5^2+gma6^2+gma7^2+gma8^2+gma9^2+gma1
0^2)/10
TE_VC := ((bta1+bta2+bta3+bta4+bta5+bta6+bta7+bta8+bta9+bta10)/10)^2
RRE_VC:=(U_pio1+U_pio2+U_pio3+U_pio4+U_pio5+U_pio6+U_pio7+U_pio8+U_pio9+U_pio
10)/10
Sum_VCs:=Trait_VC + TE_VC/1 + SFE_VC/10 + RRE_VC/(10*1)
Omega_total_coef := Trait_VC/Sum_VCs
SFE_prop := (SFE_VC/10)/Sum_VCs
TE_prop := (TE_VC/1)/Sum_VCs
RRE_prop := (RRE_VC/(10*1))/Sum_VCs
Total_error_prop := ((SFE_VC/10)+(TE_VC/1)+(RRE_VC/(10*1)))/Sum_VCs
'

Results <- lavaan(Model, data = SDQ3_appear,estimator = "MLR", orthogonal = T
RUE)
fitmeasures(Results)[c("chisq.scaled","df.scaled","cfi.scaled","tli.scaled","
rmsea.scaled")]

##  chisq.scaled     df.scaled    cfi.scaled    tli.scaled  rmsea.scaled
## 2490.84693925  170.00000000    0.89703436    0.88492075    0.08718577

mc_ci <- monteCarloCI(Results,level = 0.95)
pe <- parameterEstimates(Results)
se <- pe[pe$op == ":=", "se"]
round(cbind(mc_ci, se),3)

##                    est ci.lower ci.upper    se
## Trait_VC         1.418    1.302    1.541 0.061
## SFE_VC           0.545    0.510    0.585 0.019
## TE_VC            0.093    0.056    0.139 0.022
## RRE_VC           0.537    0.510    0.565 0.014
## Sum_VCs          1.619    1.511    1.735 0.056
## Omega_total_coef 0.876    0.845    0.900 0.014
## SFE_prop         0.034    0.031    0.037 0.002
## TE_prop          0.057    0.034    0.087 0.013
## RRE_prop         0.033    0.030    0.036 0.001
## Total_error_prop 0.124    0.100    0.155 0.014
```

4.2 Two-occasion design with wording effects

Figure S4.2. Two-occasion design with wording effects.

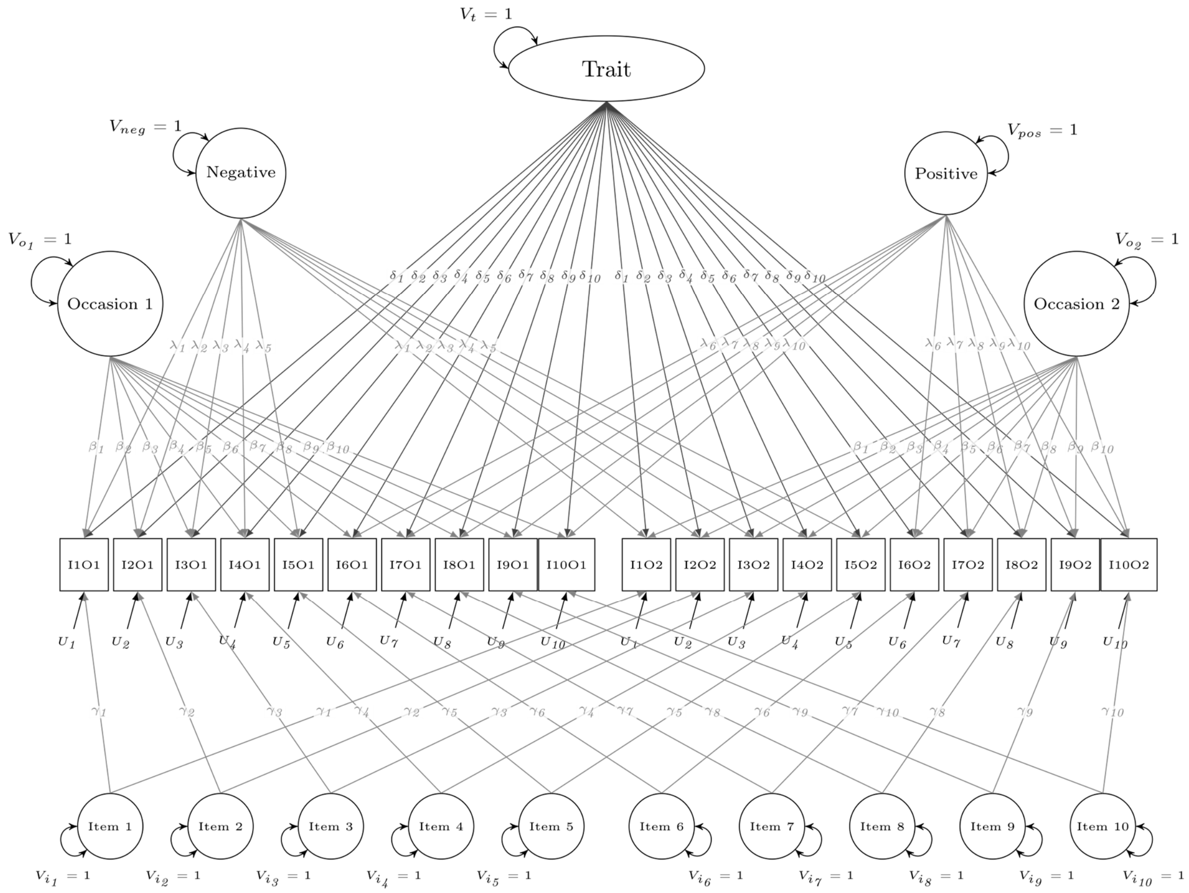


```
Model <- ' Person =~ NA*T1S12 + dlt1*T1S12 + dlt2*T1S25 + dlt3*T1S38 + dlt4*T
1S51 + dlt5*T1S64 + dlt6*T1S77+ dlt7*T1S90 + dlt8*T1S103 + dlt9*T1S116 + dlt1
0*T1S129
     + dlt1*T2S12 + dlt2*T2S25 + dlt3*T2S38 + dlt4*T2S51 + dlt5*T2S64 + dlt6*
T2S77 + dlt7*T2S90 + dlt8*T2S103 + dlt9*T2S116 + dlt10*T2S129
  Item1 =~ NA*T1S12 + gma1*T1S12 + gma1*T2S12
  Item2 =~ NA*T1S25 + gma2*T1S25 + gma2*T2S25
  Item3 =~ NA*T1S38 + gma3*T1S38 + gma3*T2S38
  Item4 =~ NA*T1S51 + gma4*T1S51 + gma4*T2S51
  Item5 =~ NA*T1S64 + gma5*T1S64 + gma5*T2S64
  Item6 =~ NA*T1S77 + gma6*T1S77 + gma6*T2S77
  Item7 =~ NA*T1S90 + gma7*T1S90 + gma7*T2S90
  Item8 =~ NA*T1S103 + gma8*T1S103 + gma8*T2S103
  Item9 =~ NA*T1S116 + gma9*T1S116 + gma9*T2S116
  Item10 =~ NA*T1S129 + gma10*T1S129 + gma10*T2S129
```

```
Occasion1 =~ NA*T1S12 + bta1*T1S12 + bta2*T1S25 + bta3*T1S38 + bta4*T1S51 + b
ta5*T1S64 + bta6*T1S77 + bta7*T1S90 + bta8*T1S103 + bta9*T1S116 + bta10*T1S12
9
Occasion2 =~ NA*T2S12 + bta1*T2S12 + bta2*T2S25 + bta3*T2S38 + bta4*T2S51 + b
ta5*T2S64 + bta6*T2S77 + bta7*T2S90 + bta8*T2S103 + bta9*T2S116 + bta10*T2S12
9


NEG =~ NA*T1S12+lmd1*T1S12+ lmd2*T1S38+lmd3*T1S64+lmd4*T1S90+ lmd5*T1S116
+lmd1*T2S12 + lmd2*T2S38 + lmd3*T2S64 + lmd4*T2S90 + lmd5*T2S116

POS =~ NA*T1S25+lmd6*T1S25 + lmd7*T1S51 + lmd8*T1S77 + lmd9*T1S103 + lmd10*T1
S129
+ lmd6*T2S25 + lmd7*T2S51 + lmd8*T2S77 + lmd9*T2S103 + lmd10*T2S129

NEG ~~ 1*NEG
POS ~~ 1*POS

## person variance
  Person ~~ 1*Person

## p x i interaction variance
  Item1 ~~ 1*Item1
  Item2 ~~ 1*Item2
  Item3 ~~ 1*Item3
  Item4 ~~ 1*Item4
  Item5 ~~ 1*Item5
  Item6 ~~ 1*Item6
  Item7 ~~ 1*Item7
  Item8 ~~ 1*Item8
  Item9 ~~ 1*Item9
  Item10 ~~ 1*Item10
## p x o interaction variance
  Occasion1 ~~ 1*Occasion1
  Occasion2 ~~ 1*Occasion2

  # 12, 25, 38, 51, 64, 77, 90, 103, 116, 129
## p x i x o interaction variance (+ error)
  T1S12 ~~ U_pio1*T1S12
  T1S25 ~~ U_pio2*T1S25
  T1S38 ~~ U_pio3*T1S38
  T1S51 ~~ U_pio4*T1S51
  T1S64 ~~ U_pio5*T1S64
  T1S77 ~~ U_pio6*T1S77
  T1S90 ~~ U_pio7*T1S90
  T1S103 ~~ U_pio8*T1S103
  T1S116 ~~ U_pio9*T1S116
  T1S129 ~~ U_pio10*T1S129
```

```
  T2S12 ~~ U_pio1*T2S12
  T2S25 ~~ U_pio2*T2S25
  T2S38 ~~ U_pio3*T2S38
  T2S51 ~~ U_pio4*T2S51
  T2S64 ~~ U_pio5*T2S64
  T2S77 ~~ U_pio6*T2S77
  T2S90 ~~ U_pio7*T2S90
  T2S103 ~~ U_pio8*T2S103
  T2S116 ~~ U_pio9*T2S116
T2S129 ~~ U_pio10*T2S129

Trait_VC := ((dlt1+dlt2+dlt3+dlt4+dlt5+dlt6+dlt7+dlt8+dlt9+dlt10)/5)^2
Neg_VC:= ((lmd1+lmd2+lmd3+lmd4+lmd5)/5)^2
Pos_VC:= ((lmd6+lmd7+lmd8+lmd9+lmd10)/5)^2
SFE_VC :=(gma1^2+gma2^2+gma3^2+gma4^2+gma5^2+gma6^2+gma7^2+gma8^2+gma9^2+gma1
0^2)/5
TE_VC := ((bta1+bta2+bta3+bta4+bta5+bta6+bta7+bta8+bta9+bta10)/5)^2
RRE_VC:=(U_pio1+U_pio2+U_pio3+U_pio4+U_pio5+U_pio6+U_pio7+U_pio8+U_pio9+U_pio
10)/5
Sum_VCs:=Trait_VC + TE_VC/1 + SFE_VC/5 + RRE_VC/(5*1) + Neg_VC + Pos_VC
Omega_total_coef := (Trait_VC + Neg_VC + Pos_VC)/Sum_VCs
Omega_hierarchical_coef := Trait_VC /Sum_VCs
NEG_prop := Neg_VC/Sum_VCs
POS_prop := Pos_VC/Sum_VCs
Total_wording_prop := NEG_prop + POS_prop
SFE_prop := (SFE_VC/5)/Sum_VCs
TE_prop := (TE_VC/1)/Sum_VCs
RRE_prop := (RRE_VC/(5*1))/Sum_VCs
Total_error_prop := ((SFE_VC/5)+(TE_VC/1)+(RRE_VC/(5*1)))/Sum_VCs'

make_item_code <- function(i) {
  item <- c(1, 3, 5, 7, 9, 2, 4, 6, 8, 10)[i]
  if (i <= 5) {
    k <- i
    wording <- paste0("lmd", k)
    label <- "Neg"
  } else {
    k <- i
    wording <- paste0("lmd", k)
    label <- "Pos"
  }
  c(
    paste0("sum_", i, " := dlt", item, "^2 + ", wording, "^2 + bta", item, "^
2 + gma", item, "^2 + U_pio", item),
    paste0("Trait_", i, " := dlt", item, "^2/sum_", i),
    paste0(label, "_", i, " := ", wording, "^2/sum_", i),
    paste0("SFE_", i, " := gma", item, "^2/sum_", i),
    paste0("TE_", i, " := bta", item, "^2/sum_", i),
    paste0("RRE_", i, " := U_pio", item, "/sum_", i),
    paste0("TotalError_", i, " := SFE_", i, " + TE_", i, " + RRE_", i)
```

```
  )
}
item_level <- paste(unlist(lapply(1:10, make_item_code)), collapse = "\n")


Results <- lavaan(paste(Model, item_level, sep = "\n"), data = SDQ3_appear,es
timator = "MLR", orthogonal = TRUE)
fitmeasures(Results)[c("chisq.scaled","df.scaled","cfi.scaled","tli.scaled","
rmsea.scaled")]
```

```
## chisq.scaled    df.scaled   cfi.scaled   tli.scaled rmsea.scaled
## 824.73484151 160.00000000   0.97050868   0.96497905   0.04809623
```

```
mc_ci <- monteCarloCI(Results,level = 0.95)
pe <- parameterEstimates(Results)
se <- pe[pe$op == ":=", "se"]
round(cbind(mc_ci, se),3)
```

```
##                           est ci.lower ci.upper    se
## Trait_VC                5.266    4.813    5.732 0.234
## Neg_VC                  0.296    0.245    0.351 0.027
## Pos_VC                  0.255    0.211    0.302 0.023
## SFE_VC                  0.701    0.656    0.759 0.027
## TE_VC                   0.282    0.211    0.363 0.038
## RRE_VC                  1.034    0.983    1.086 0.026
## Sum_VCs                 6.446    6.009    6.901 0.225
## Omega_total_coef        0.902    0.888    0.915 0.007
## Omega_hierarchical_coef 0.817    0.794    0.837 0.011
## NEG_prop                0.046    0.037    0.055 0.005
## POS_prop                0.040    0.032    0.048 0.004
## Total_wording_prop      0.085    0.074    0.099 0.006
## SFE_prop                0.022    0.020    0.024 0.001
## TE_prop                 0.044    0.033    0.057 0.006
## RRE_prop                0.032    0.029    0.035 0.001
## Total_error_prop        0.098    0.085    0.112 0.007
## sum_1                   2.174    2.013    2.351 0.086
## Trait_1                 0.683    0.632    0.725 0.024
## Neg_1                   0.002    0.000    0.007 0.002
## SFE_1                   0.098    0.073    0.126 0.014
## TE_1                    0.041    0.016    0.078 0.016
## RRE_1                   0.176    0.145    0.207 0.016
## TotalError_1            0.315    0.274    0.364 0.023
## sum_2                   2.755    2.587    2.937 0.089
## Trait_2                 0.664    0.620    0.704 0.021
## Neg_2                   0.046    0.028    0.067 0.010
## SFE_2                   0.076    0.060    0.094 0.009
## TE_2                    0.056    0.033    0.085 0.013
## RRE_2                   0.158    0.132    0.183 0.013
## TotalError_2            0.290    0.259    0.325 0.017
## sum_3                   3.714    3.563    3.893 0.084
## Trait_3                 0.293    0.252    0.333 0.021
```

```
## Neg_3                      0.305    0.248    0.364 0.030
## SFE_3                      0.149    0.103    0.203 0.025
## TE_3                       0.016    0.005    0.032 0.007
## RRE_3                      0.237    0.211    0.262 0.013
## TotalError_3               0.402    0.353    0.456 0.026
## sum_4                      2.351    2.227    2.497 0.069
## Trait_4                    0.390    0.349    0.431 0.021
## Neg_4                      0.033    0.017    0.054 0.009
## SFE_4                      0.351    0.316    0.386 0.018
## TE_4                       0.011    0.002    0.028 0.007
## RRE_4                      0.214    0.191    0.236 0.012
## TotalError_4               0.576    0.537    0.617 0.020
## sum_5                      3.371    3.229    3.546 0.079
## Trait_5                    0.439    0.396    0.479 0.021
## Neg_5                      0.275    0.217    0.333 0.030
## SFE_5                      0.050    0.015    0.106 0.023
## TE_5                       0.027    0.014    0.045 0.008
## RRE_5                      0.208    0.183    0.231 0.012
## TotalError_5               0.285    0.243    0.341 0.025
## sum_6                      1.854    1.724    1.996 0.069
## Trait_6                    0.741    0.704    0.772 0.017
## Pos_6                      0.002    0.000    0.007 0.002
## SFE_6                      0.043    0.023    0.067 0.011
## TE_6                       0.038    0.023    0.058 0.009
## RRE_6                      0.177    0.148    0.205 0.014
## TotalError_6               0.258    0.225    0.295 0.018
## sum_7                      2.865    2.717    3.035 0.080
## Trait_7                    0.424    0.376    0.469 0.024
## Pos_7                      0.357    0.308    0.405 0.025
## SFE_7                      0.027    0.007    0.059 0.013
## TE_7                       0.029    0.016    0.044 0.007
## RRE_7                      0.164    0.143    0.184 0.010
## TotalError_7               0.220    0.189    0.257 0.017
## sum_8                      2.718    2.564    2.891 0.083
## Trait_8                    0.576    0.529    0.620 0.023
## Pos_8                      0.208    0.172    0.245 0.019
## SFE_8                      0.033    0.017    0.054 0.009
## TE_8                       0.027    0.012    0.049 0.010
## RRE_8                      0.156    0.132    0.179 0.012
## TotalError_8               0.216    0.189    0.247 0.015
## sum_9                      1.993    1.850    2.149 0.077
## Trait_9                    0.581    0.537    0.621 0.021
## Pos_9                      0.006    0.001    0.017 0.004
## SFE_9                      0.201    0.163    0.239 0.020
## TE_9                       0.024    0.013    0.040 0.007
## RRE_9                      0.188    0.166    0.212 0.012
## TotalError_9               0.413    0.370    0.457 0.022
## sum_10                     3.645    3.463    3.845 0.097
## Trait_10                   0.323    0.281    0.364 0.021
## Pos_10                     0.237    0.201    0.277 0.019
```

```
## SFE_10                    0.244    0.211    0.278 0.017
## TE_10                     0.012    0.004    0.023 0.005
## RRE_10                    0.185    0.162    0.207 0.012
## TotalError_10             0.440    0.401    0.481 0.021
```

Abbreviations used throughout the supplement

SDQ-III: Self-Description Questionnaire-III

VC: Variance component

Coef: Coefficient

Prop: Proportion of explained observed score variance

Trait: The construct being measured. In the present examples, perceptions of Physical Appearance serve as the construct of interest.

NEG or neg: Negative wording

POS or pos: Positive wording

SFE : Specific-factor error

TE: Transient error

RRE: Random-response error

Omega hierarchical coefficient (Omega_heirarchical_coef): In the analyses reported here, this coefficient represents the proportion of observed score variance accounted for by the trait alone.

Omega total coefficient (Omega_total_coef): In the analyses reported here, this coefficient represents the proportion of observed score variance accounted for by the trait and item wording effects combined.

SE or se: Standard error for the parameter of interest

dlt1, dlt2… = Trait loadings (deltas)

lmd1, lmd2… = Negative and positive wording loadings (lambdas)

bta1, bta2… = Occasion loadings (betas)

gma1, gma2… = Item loadings (gammas)

References

Jorgensen, T. D., Pornprasertmanit, S., Schoemann, A. M., & Rosseel, Y. (2025). *semTools: Useful tools for structural equation modeling*. R package version 0.5-7. Retrieved from https://CRAN.R-project.org/package=semTools

Marsh, H.W. (1992). Self-Description Questionnaire (SDQ) III: A theoretical and empirical basis for the measurement of multiple dimensions of late adolescent self-concept. An interim test manual and research monograph; University of Western Sydney: Macarthur, Australia.

Rosseel, Y. (2012). lavaan: An R Package for Structural Equation Modeling. *Journal of Statistical Software*, *48*(2), 1-36. https//doi.org/10.18637/jss.v048.i02

Rosseel, Y., Jorgensen, T. D., & De Wilde, L. (2025). *lavaan*: *Latent Variable Analysis*. R package version 0.6-20. https://cran.r-project.org/web/packages/lavaan/lavaan.pdf
